# Translational potential of GMP-grade human umbilical cord-derived mesenchymal stem cells (UC-MSCs) in traumatic spinal cord injury: a preclinical study in rat

**DOI:** 10.1186/s12967-026-08373-x

**Published:** 2026-06-10

**Authors:** Zhibo Han, Hao Yu, Wenjing Du, Yuchen Gao, Kai Pan, Honghong Jia, Meng Zhao, Zhe Wei, Shuling Yan, Youwei Wang, Zongjin Li, Guangjian Ni

**Affiliations:** 1 Institute of Medical Engineering & Translational Medicine, Tianjin University, Tianjin, 300072 China; 2 Tianjin Key Laboratory of Engineering Technologies for Cell Pharmaceutical, National Engineering Research Center of Cell Products, AmCellGene Co., Ltd, Tianjin, 300457 China; 3 School of Medicine, Nankai University, Tianjin, 300071 China; 4 State Key Laboratory of Experimental Hematology, Institute of Hematology & Blood Diseases Hospital, Chinese Academy of Medical Sciences & Peking Union Medical College, Tianjin, 300020 China

## Abstract

**Background:**

Traumatic spinal cord injury (SCI) often results in irreversible motor, sensory, and autonomic dysfunction, with limited effective treatment options currently available. Human umbilical cord-derived mesenchymal stem cells (UC-MSCs) represent a promising therapeutic approach due to their immunomodulatory, neuroprotective, and regenerative properties. However, the lack of comprehensive efficacy data using GMP-grade cells, uncertainty regarding optimal dosing, and incomplete understanding of their mechanisms have hindered clinical translation.

**Methods:**

A T10-level SCI model was established in 50 Sprague‒Dawley rats using Allen’s weight-drop method. The animals were randomly divided into five groups: Sham, Model, Solvent, Low-dose (1 × 10⁷ cells/kg), and High-dose (3 × 10⁷ cells/kg). GMP-grade human UC-MSCs were administered intravenously on post-injury days 3 and 7. A comprehensive evaluation was performed using BBB scoring for locomotor function, MRI for lesion volume assessment, histopathological examination (H&E and Nissl staining), ELISA for serum cytokine quantification, immunofluorescence for GFAP and GAP-43 expression, single-cell RNA sequencing (scRNA-seq) of peripheral blood mononuclear cells (PBMCs), in *vivo* UC-MSCs tracking, and sRNA sequencing of human UC-MSC-derived exosomes.

**Results:**

The high-dose UC-MSC treatment demonstrated significant therapeutic effects, including: improved hindlimb motor function (BBB scores from days 7 to 21), reduced spinal cord lesion volume, attenuated pathological damage (decreased cavity formation, preserved neuronal morphology, increased neuron density), and suppressed acute inflammation (reduced TNF-α and IL-6 levels). Additionally, high-dose treatment decreased astrocyte activation (reduced GFAP expression), enhanced neuronal plasticity (increased GAP-43), modulated immune cell populations (increased naïve CD4⁺/CD8⁺ T cells, decreased memory B cells), and downregulated SCI-activated genes (including Kras and Nfkb1). In vivo tracking revealed initial pulmonary accumulation of UC-MSCs followed by clearance within 3 days. sRNA sequencing of human UC-MSC-derived exosomes identified several human-derived miRNAs (hsa-miR-21-5p, hsa-let-7a-5p, hsa-miR-10b-5p, hsa-miR-451a, and hsa-miR-10a-5p) potentially involved in the repair process.

**Conclusions:**

GMP-grade human UC-MSCs exert therapeutic effects in SCI through multiple mechanisms, including anti-inflammatory actions, inhibition of glial scarring, neuroprotection, and immune modulation. The high-dose regimen (3 × 10⁷ cells/kg) demonstrated superior efficacy across functional, structural, and molecular endpoints. This study provides critical preclinical evidence supporting the clinical application of UC-MSCs for SCI treatment and elucidates their underlying therapeutic mechanisms.

**Supplementary Information:**

The online version contains supplementary material available at 10.1186/s12967-026-08373-x.

## Introduction

Traumatic spinal cord injury (SCI) is a severe central nervous system trauma and is one of the disease types with the highest incidence of long-term disability [1]. It is caused mainly by related injuries such as traffic accidents, falls, sports, or violence [2]. A total of 759,302 patients with traumatic SCI have been reported in China, with 66,374 new cases added each year [3, 4]. SCI often results in temporary or permanent neurological deficits in the body below the level of injury, including motor, sensory, and autonomic dysfunction; loss or alteration of sensations such as pain, temperature, and touch; limb paralysis; hyperactive or spasmodic reflexes; loss of bowel or bladder sphincter control; and autonomic dysfunction [5]. The initial pathological manifestations of SCI typically include damage to neurons and oligodendrocytes in the central region of the injury, rupture of blood vessels, and disruption of the blood‒spinal cord barrier. This is subsequently followed by secondary injury cascades, including local ischemia and inflammatory responses, which cause further tissue deterioration [1]. Severe inflammation is a major obstacle in the acute phase of SCI [6]. At present, the known clinical treatment methods for SCI mainly include surgical intervention, drug therapy, and rehabilitation [5]. However, these treatments typically fail to effectively restore neurological functions in patients. Axons that serve in the spinal cord are often thought to be incapable of long-distance regeneration [2, 7, 8]. Once the axon is damaged, its poor regenerative ability prevents it from passing through the damaged area, irreversibly impairing the transmission of motor and sensory information between the brain and the distal spinal cord, resulting in neurological dysfunction that becomes permanent damage and leads to lifelong disability in patients with SCI [9].

Mesenchymal stem cells (MSCs) are multipotent stem cells derived from the mesoderm that can be isolated from various human tissues and organs, such as bone marrow, adipose tissue, and perinatal sources such as the umbilical cord, placenta, and amniotic membrane [10]. Over the past few decades, MSCs have shown great potential in the treatment of SCI because of their self-renewal capacity, multidirectional differentiation potential, low immunogenicity, strong proliferation ability, and multiple biological activities, such as anti-inflammatory effects, immune modulation, angiogenesis, trophic effects and paracrine effects [2, 11–15]. MSCs can migrate to damaged neuronal sites, exert immune regulation, reduce inflammatory responses, inhibit mitochondrial oxidative stress, and exert neuroprotective effects, including neurotrophic factor secretion (such as nerve growth factor and brain-derived neurotrophic factor), and endogenous neural stem cell stimulation [15–18]. In addition, MSCs have been reported to promote neurogenesis, differentiation into neuronal progenitor cells or mature neuronal cells [19]. In addition, MSCs have been shown to reduce neuronal apoptosis and promote nerve regeneration in neurological diseases [18, 20]. However, an increasing number of preclinical and clinical trials have shown that MSCs are safe and effective in the treatment of neurodegenerative and neurotraumatic diseases, such as Alzheimer’s disease (AD), multiple sclerosis (MS), spinal cord injury (SCI), and stroke [18, 21–25]. However, the current clinical drug research on the use of MSCs for the treatment of SCI is not complete, and to date, no MSC-based drug for SCI treatment has been approved for marketing worldwide. Moreover, the therapeutic mechanism of MSCs in the treatment of SCI remains unclear [6, 25, 26].

In this study, we transplanted good manufacturing practice (GMP)-grade human umbilical cord-derived mesenchymal stem cells (UC-MSCs) into rats with traumatic SCI to investigate their therapeutic efficacy and explore the optimal intravenous dosing regimen for UC-MSCs by comparing two different cell doses, thereby providing important experimental evidence for future clinical translational studies. In addition, single-cell transcriptome analysis was performed to determine the potential therapeutic mechanisms of UC-MSCs for SCI treatment.

## Methods and materials

### Experimental design, grouping, and UC-MSCs transplantation

**Fig. 1:**
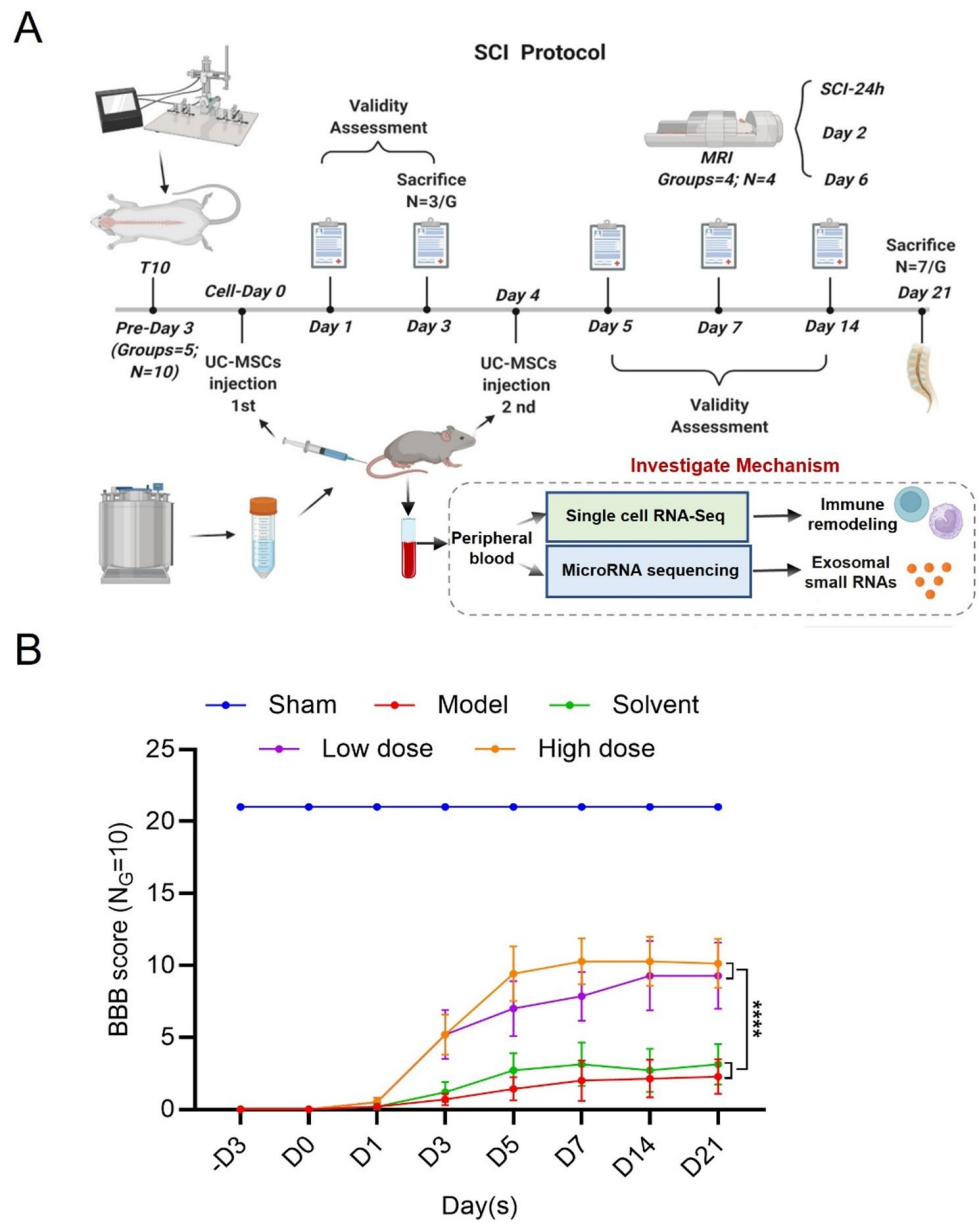
The BBB scores of rats with spinal cord injury (SCI) were significantly improved after intravenous UC-MSCs administration. (**A**) Schematic representation of the study design and experimental procedure. (**B**) BBB locomotor scores of Sham, Model, Solvent, Low-dose, and High-dose UC-MSC groups recorded at indicated time points (*n* = 10). Statistical analysis was performed using one-way ANOVA followed by Tukey’s post-hoc test; ^****^p < 0.0001

The off-the-shelf product, UC-MSCs, is manufactured under GMP conditions and has received clinical trial approval (CXSL1800117) from National Medical Products Administration (NMPA) of China in the treatment of diabetic foot ulcers. The product has completed Phase I clinical trials for treating diabetic foot ulcers, demonstrating good safety. All animal experiments were carried out in accordance with the standard operating procedures (SOPs) of Tianjin MEDTECH Co., Ltd, the Guide for the Care and Use of Laboratory Animals, 8th Edition, and US Department of Agriculture Animal Welfare Regulations (Public Law 99–198). Fifty 8-week-old male Sprague‒Dawley rats (100–120 g) were acclimatized for one week under standard specific pathogen-free conditions (temperature: 20 ± 2 °C; humidity: 45–55%; 12/12 h light/dark cycle) before being randomly assigned to five groups (*n* = 10): the sham group, model group, solvent group, high-dose group (3 × 10⁷ cells/kg), and low-dose group (1 × 10⁷ cells/kg). The overall experimental design is schematically illustrated in Fig. 1. Spinal cord injury (SCI) was induced at the T10 level *via* Allen’s weight‒drop method [27], except in the sham groups, which received laminectomy only. Successful model establishment was confirmed by immediate spastic tail movement and hindlimb retraction, followed by persistent flaccid paralysis post-anesthesia. On days 3 and 7 post-injury, the respective treatments were administered *via* tail vein injection at a volume of 400 µL.

### Behavioral assessment

Hindlimb locomotor function was assessed *via* the Basso-Beattie-Bresnahan (BBB) locomotor rating scale (scores of 0–21, representing complete paralysis to normal locomotion, respectively) [28, 29]. Following a 5-minute acclimation period in an open field, each rat was evaluated by two independent observers blinded to the experimental groups. The final score for each animal was calculated as the mean from both hindlimbs. Assessments were conducted at baseline (-D3), pre-treatment (D0), and on days 1, 3, 5, 7, 14, and 21 post-treatments.

### Gross morphology and MRI detection

At 21 days post-treatment, the rats were euthanized, and the spinal cord segment encompassing the injury epicenter (approximately 1.5 cm) was carefully harvested, rinsed with saline, and immediately evaluated by blinded observers for features such as color, texture, cyst formation, and tissue integrity. Magnetic resonance imaging (MRI) was performed via a micro-MRI 9.4T scanner (Bruker, micro-MRI 9.4T, USA) to longitudinally assess the lesion site in vivo. Scans were conducted at 24 h post-SCI and at 2 and 6 days post-SCI (*n = 4* per time point) for the four groups (sham-, model-, high- and low-dose groups). For each animal, two representative T2-weighted sagittal images were selected, and regions of interest (ROIs) exhibiting hypointense and hyperintense signals were delineated via the Freehand tool to quantify and compare the lesion volume.

### Hematoxylin‒eosin and Nissl staining

Spinal cord samples from each group were fixed, routinely processed through a graded alcohol series for dehydration, and subsequently embedded in paraffin using a Leica EG1150C tissue embedding machine. Serial sections were prepared with a Leica RM2245 microtome. For histological evaluation, the sections were subjected to either hematoxylin and eosin (H&E) or Nissl staining. The H&E staining procedure involved staining with hematoxylin, washing with distilled water, differentiation in 1% hydrochloric acid to remove background staining, and counterstaining with eosin. Nissl staining was performed via Nissl staining solution (Beyotime, C0117) at 37–50 °C for 3–10 min, followed by washing in distilled water and 95% ethanol and clearing in xylene. All stained sections were mounted with neutral resin and imaged using a Leica ICC50W microscope for assessment of general cellular morphology and neuronal integrity.

### Measurement of serum cytokine levels

To assess the systemic inflammatory and repair responses following cell therapy, peripheral blood samples were collected from the rats in each group on days 1, 3, and 7 post-treatment. After centrifugation, the serum was aliquoted and stored at -80 °C until analysis. The levels of interleukin-1β (IL-1β), tumor necrosis factor-α (TNF-α), and interleukin-6 (IL-6), were quantified via commercial rat-specific ELISA kits (FANKEW, Shanghai, China) in accordance with the manufacturer’s protocols. The absorbance was measured at 450 nm via a microplate reader (BioTek BTI-ELX800, USA), and the cytokine concentrations were determined via Prism 10 software.

### Immunofluorescence staining

Tissue repair at the injured spinal cord site in rats with spinal cord injury (SCI) was evaluated. On post-cell therapy day 21, the rats in each group were euthanized, and their injured spinal cord regions, along with adjacent tissues, were dissected, stored at -80 °C, subsequently dehydrated in 30% sucrose, embedded in O.C.T. compound, and finally cut into 6-µm frozen sections. The sections were treated with citrate antigen retrieval buffer, blocked with 3% bovine serum albumin for 1 h, and incubated at 37 °C for 1 h with primary antibodies (GFAP rabbit mAb, 1:2000, ABclonal, China, A19058; or GAP-43 rabbit pAb, 1:2000, ABclonal, China, A16857), followed by three 5-min washes with PBS. The corresponding secondary antibodies (HRP Donkey Anti-Rabbit IgG (H + L), 1:2000, ABclonal, China, AS038; or FITC Goat Anti-Rabbit IgG (H + L), 1:2000, ABclonal, China, AS011) were then incubated at 37 °C in the dark for 1 h, followed by another three 5-min washes with PBS. Nuclei were stained with DAPI at room temperature for 5 min; after three additional PBS washes, the sections were imaged *via* a fluorescence microscope (Olympus, LX7, Japan; 400×). Specific protein expression levels are indicated by the mean fluorescence intensity (analyzed *via* ImageJ, NIH, USA), which was calculated as follows: Mean = total fluorescence intensity (IntDen) of the area/area of the region.

### Principal component analysis (PCA)

Principal component analysis (PCA) was employed to delineate the internal structure of the current multivariate dataset, which was represented as a set of coordinates in the corresponding high-dimensional data space, which is consistent with the methodologies reported by our research group and other investigators [13]. Additionally, the effects of different treatment groups and their correlations with other characteristics were statistically determined *via* XLSTAT software (Addinsoft, New York, USA) and the OmicShare tools at http://www.omicshare.com/tools.

### Single-cell RNA sequencing of PBMCs from SCI rats in the different groups

The immune cells were isolated from the peripheral blood samples of the rats in the sham, model, and high-dose groups on post-cell therapy day 21. In brief, 10,000 cells were targeted after reverse transcription, cDNA amplification, and library construction were performed with the 10X Gen omics Chromium Single Cell 3′ Reagent Kit (v2) according to the manufacturer’s instructions. The cells were processed for scRNA-seq on the 10X Genomics Chromium platform (10X Genomics).

### In vivo tracking of UC-MSCs products

In vivo tracking of UC-MSCs products to assess their spatiotemporal distribution in rats with SCI was performed using the IVIS Lumina II system (Xenogen Corporation, Hopkinton, MA), with bioluminescence imaging (BLI) signals quantified as photons/s/cm²/sr as the readout. Briefly, a lentiviral vector encoding dual reporter genes (firefly luciferase, Fluc; enhanced green fluorescent protein, eGFP) was constructed (pLV-Fluc-eGFP), and UC-MSCs were transduced with this lentivirus at a multiplicity of infection (MOI) of 10, with transduction efficiency verified by flow cytometry (BD FACSAria III) at 72 h post-transduction. For the in vivo BLI assay, anesthetized rats were intraperitoneally injected with the Fluc substrate D-Luciferin (33 µl/mg; Cayman Chemical, USA), and BLI images were acquired at 1 h, 2 h, 4 h, 8 h, day 1 (D1), and day 3 (D3) post-UC-MSC administration *via* Living Image Software (Xenogen Corporation, Hopkinton, MA) with a 10-minute exposure time per image, with signal intensity further analyzed using the same software. All experimental procedures were conducted in strict compliance with the applicable institutional and/or national guidelines for the care and use of laboratory animals.

### Human-derived exosome sRNA sequencing analysis

Rat peripheral blood samples were collected 24 hours before and after tail vein injection. Total RNA was extracted and quality-verified prior to library construction *via* the Small RNA-seq Lib Prep Kit. Leveraging the unique 5’ phosphate and 3’ hydroxyl groups of sRNAs, adapters were ligated directly to total RNA-derived sRNAs, which were reverse-transcribed into cDNA, amplified *via* PCR, and purified *via* PAGE to generate cDNA libraries. Libraries were initially quantified with a Qubit 2.0 (diluted to 1 ng/µl), validated for insert size *via* an Agilent 2100, and accurately quantified *via* qPCR to ensure an effective concentration of > 2 nM. The qualified libraries were pooled by concentration and target data volume for Illumina SE50 sequencing. Bioinformatic analyses included profiling human-derived microRNA (miRNA) expression changes, predicting target genes *via* TargetScan, performing GO enrichment analysis *via* OmicShare tools, and constructing/visualizing protein‒pathway interaction networks with STRING and Cytoscape software.

### Statistical analysis

All experimental data were expressed as the mean ± standard deviation (SD) unless otherwise specified. Sample size (*n*) for each experiment is indicated in the figure legends and corresponding subsections. Comparisons between two groups were performed using the unpaired Student’s t-test, and multiple group comparisons were conducted using one-way analysis of variance (ANOVA) followed by Tukey’s post-hoc test. Principal component analysis (PCA) was performed using XLSTAT software (Addinsoft, New York, USA) and OmicShare tools. Statistical analysis for omics data (scRNA-seq, exosomal sRNA sequencing) is detailed in their respective subsections. Statistical significance was defined as ^*^ p < 0.05, ^**^ p < 0.01, ^***^ p < 0.001, ^****^ p < 0.0001; “*ns*” indicates no significant difference (*p > 0.05*). All statistical analyses were performed using GraphPad Prism 10 (GraphPad Software, USA) and R 4.3.1 software.”

## Results

### Administration of UC-MSCs after SCI significantly improved the BBB score in rats

The experimental timeline is depicted in Fig. 1A. Spinal cord injury (SCI) was induced on Day 3. Treatments were administered on specific days, and various assessments were conducted throughout the experiment until the animals were sacrificed on Day 21. These assessments included BBB score and body weight, scRNA-seq/small RNA-seq, magnetic resonance imaging (MRI), ELISA, immunofluorescence/immunohistochemical staining, and hematoxylin and eosin (H&E)/Nissl staining. To evaluate the therapeutic efficacy of UC-MSCs, BBB scoring was performed at multiple time points (Fig. 1B). The sham groups had a BBB score of 21. After SCI induction, all injured rats had a BBB score of 0, indicating complete hindlimb paralysis. Compared with the model groups, the solvent groups showed no significant improvement in the BBB score from Day 3 to D21 (*p > 0.05*). In contrast, the BBB scores of the rats in the high-dose and low-dose groups were significantly higher than those of the model and solvent groups from day 7 to day 21 post-treatment (^****^P < 0.01). The trajectory of body weight change was consistent with BBB score (Fig. S1). This improvement in hindlimb locomotor function and weight recovery suggests that UC-MSCs may promote neurological recovery in SCI rats.

### Administration of UC-MSCs subsequent to SCI remarkably facilitated the recovery of general behavioral functions in rats

**Fig. 2:**
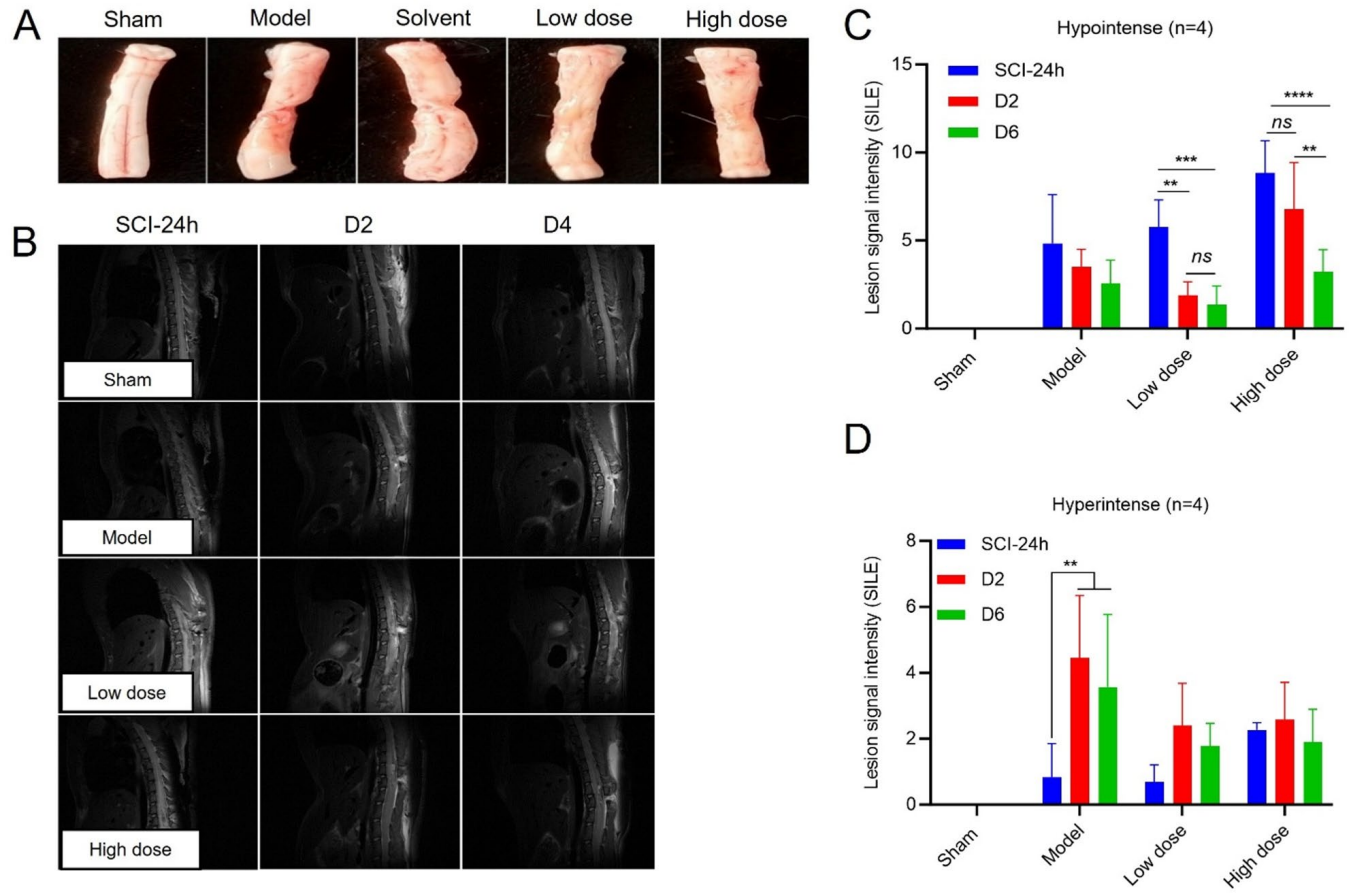
The general behavioral functions of rats with spinal cord injury (SCI) are significantly improved after intravenous administration of UC-MSCs. (**A**) Representative gross morphology of the spinal cord injury site in different treatment groups at 21 days after cell therapy (*n = 10*). (**B**) Representative T2-weighted MR images of the spinal cord in different treatment groups before cell therapy and after two rounds of cell therapy (*n* = 4 rats per time point). (**C**) Analysis of hypointense values in MR images of different treatment groups before cell therapy and after two rounds of cell therapy (*n = 4*). (**D**) Analysis of hyperintense values in MR images of different treatment groups before cell therapy and after two rounds of cell therapy (*n = 4*). Statistical analysis was performed using one-way ANOVA followed by Tukey’s post-hoc test; ^**^p < 0.01, ^***^p < 0.001, ^****^p < 0.0001, ns = not significant (*P* > 0.05)

The spinal cord samples were harvested, and the gross morphology is shown in Fig. 2A. Compared with those in the sham groups, the spinal cord tissues in the model and solvent groups presented significant damage gaps. Particularly in the model groups, the spinal cord bundle was almost completely severed in the middle, with evident redness, swelling, and congestion at the injury site. In contrast, after treatment with high and low doses of UC-MSCs, traces of spinal cord damage were still visible, while no significant redness but apparent fiber recovery was observed. Considering the high sensitivity of MRI for detecting occult bone injuries, the therapeutic effects of intravenous infusion of cells in SCI rats should be analyzed. Spinal cord MR images of each group are presented in Fig. 2B. The lesion signal intensity (SILE), encompassing hypointense and hyperintense components, was quantified and analyzed, and the results are shown in Fig. 2C, D. In T2-weighted MRI of spinal cord injury, hypointense signals typically indicate the formation of cystic cavitation and necrotic tissue at the injury site, while hyperintense signals reflect acute pathological changes including tissue edema, hemorrhage, and inflammatory infiltration at the SCI epicenter. The quantification of these two signal types allows for objective assessment of spinal cord lesion severity and repair efficiency.

Hypointense signals on MRI typically indicate cavity formation at the SCI site, whereas hyperintense signals often reflect lesion edema and bleeding. Compared with those in the sham group, both signal types were significantly greater in post-SCI rats, thus confirming successful SCI model establishment.

As shown in Fig. 2C, the hypointense values in the model group slightly decreased over time. In contrast, after two rounds of UC-MSCs treatment, these values were significantly lower in both the high- and low-dose groups than in the control group, indicating that intravenous UC-MSCs can effectively reduce the spinal cord lesion area. Specifically, in the low-dose groups, the baseline value was 5.76 ± 1.54, which decreased significantly to 1.88 ± 0.78 after the first treatment (*p < 0.01*) and further to 1.34 ± 1.08 after the second treatment (^**^p < 0.01), although no significant difference was observed between the two treatments (*p > 0.05*). In the high-dose groups, the baseline hypointense value was 8.84 ± 1.83, which decreased to 6.77 ± 2.66 after the first treatment and further to 3.20 ± 1.28 after the second treatment (^**^p < 0.01), with a significant difference between the two treatments (^**^p < 0.01). As shown in Fig. 2D, in the model group, the hyperintense values in the rats significantly increased from 0.84 ± 1.02 to 4.45 ± 1.89 and 3.57 ± 2.20 after two rounds of PBS treatment (^**^p < 0.01). However, in the rats in the high- and low-dose groups, the hyperintense values did not significantly increase compared with those before treatment (*p > 0.05*), indicating that UC-MSCs treatment does not exacerbate edema or bleeding associated with SCI.

### Administration of UC-MSCs following SCI markedly promoted the recovery of pathological structures in rats

**Fig. 3:**
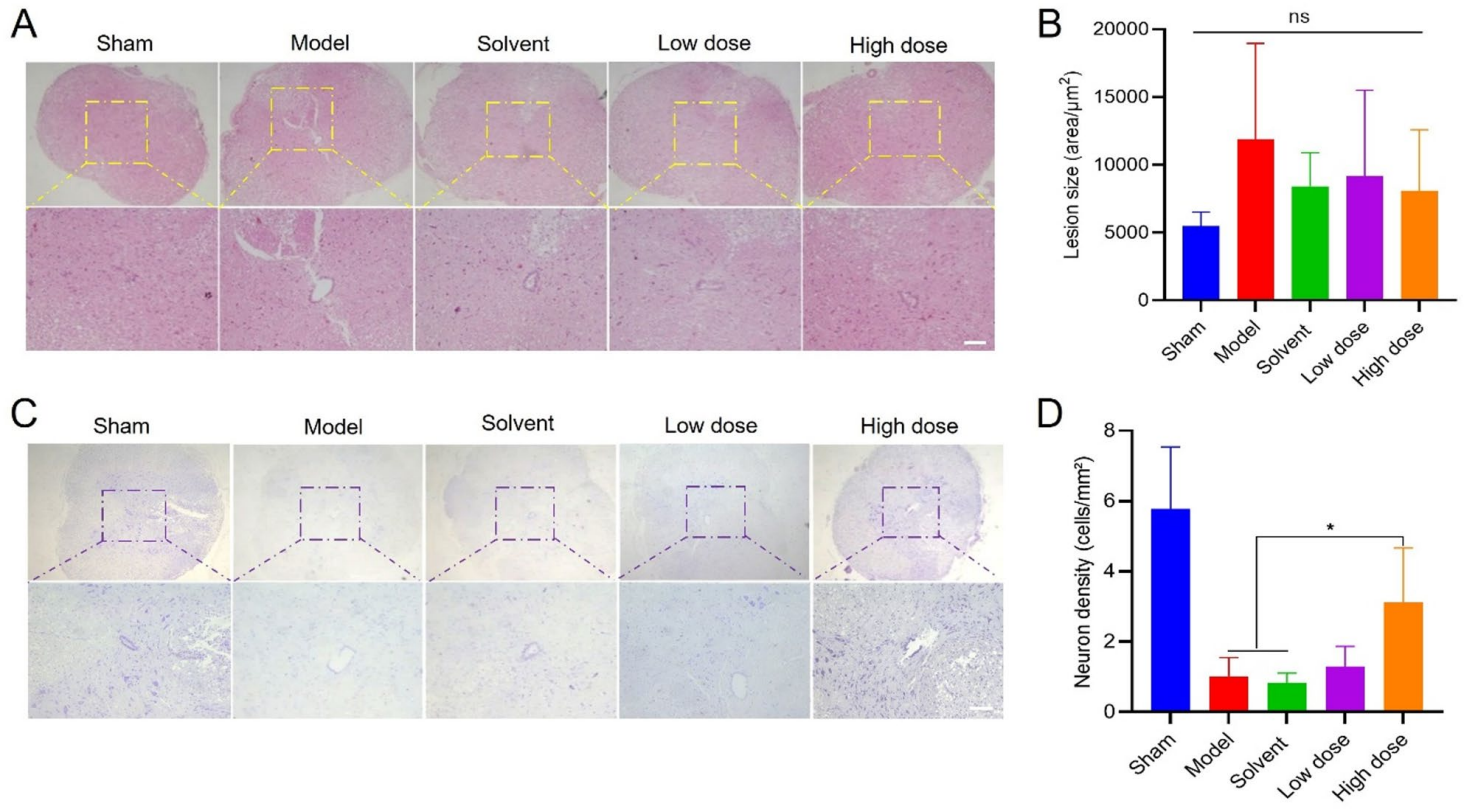
The intravenous administration of UC-MSCs significantly improved the pathological morphology of the spinal cord in rats with spinal cord injury (SCI). (**A**) HE staining of spinal cord tissue sections from different treatment groups at 21 days after cell therapy. (**B**) Quantitative analysis of the lesion area on the basis of the HE staining results. (**C**) Nissl staining of spinal cord tissue sections from different treatment groups at 21 days after cell therapy. (**D**) Quantitative analysis of the lesion area on the basis of the Nissl staining results. Statistical analysis was performed using one-way ANOVA followed by Tukey’s post-hoc test; ^*^P < 0.05, *ns* = not significant (*P* > 0.05)

To assess the therapeutic effects of UC-MSCs, histological analyses, including HE staining and Nissl staining, were conducted on spinal cord tissues from different treatment groups, as shown in Fig. 3A and C. In the sham group, HE staining revealed intact and clearly demarcated gray and white matter structures of the spinal cord; neurons in the gray matter and nerve fibers in the white matter appeared normal, with clear and abundant Nissl bodies in neuronal cells, and no significant changes in glial cells were observed. However, in the model and solvent groups, some gray matter neurons were shrunken, with nuclei disappearing or becoming degenerated and swollen, staining becoming lighter, and Nissl bodies unclear or absent. The proliferation of astrocytes and microglia causes swelling and degeneration of white matter nerve fibers, leading to focal or patchy necrosis and “cavities”, along with visible lymphocytes and macrophages at injury sites and evident vascular congestion. In contrast, especially in the high-dose groups, inflammatory cell infiltration at the injury site was significantly reduced; the morphology of gray matter neurons and white matter nerve fibers tended to be normal, and Nissl bodies in neuronal cells were clearly visible. Additionally, quantitative analysis of the lesion area (Fig. 3B) revealed no significant difference between the model and solvent groups, whereas the lesion areas in the low-dose and high-dose groups were smaller (although statistical significance was not fully reached). In terms of neuron density (Fig. 3D), the model groups presented markedly lower neuron density than the sham group did; however, the high-dose groups presented a significant increase in neuron density compared with the model group (*p < 0.05), indicating that it promoted neuronal survival or regeneration.

### Administration of UC-MSCs after SCI remarkably facilitated the recovery of biological functions in rats

**Fig. 4:**
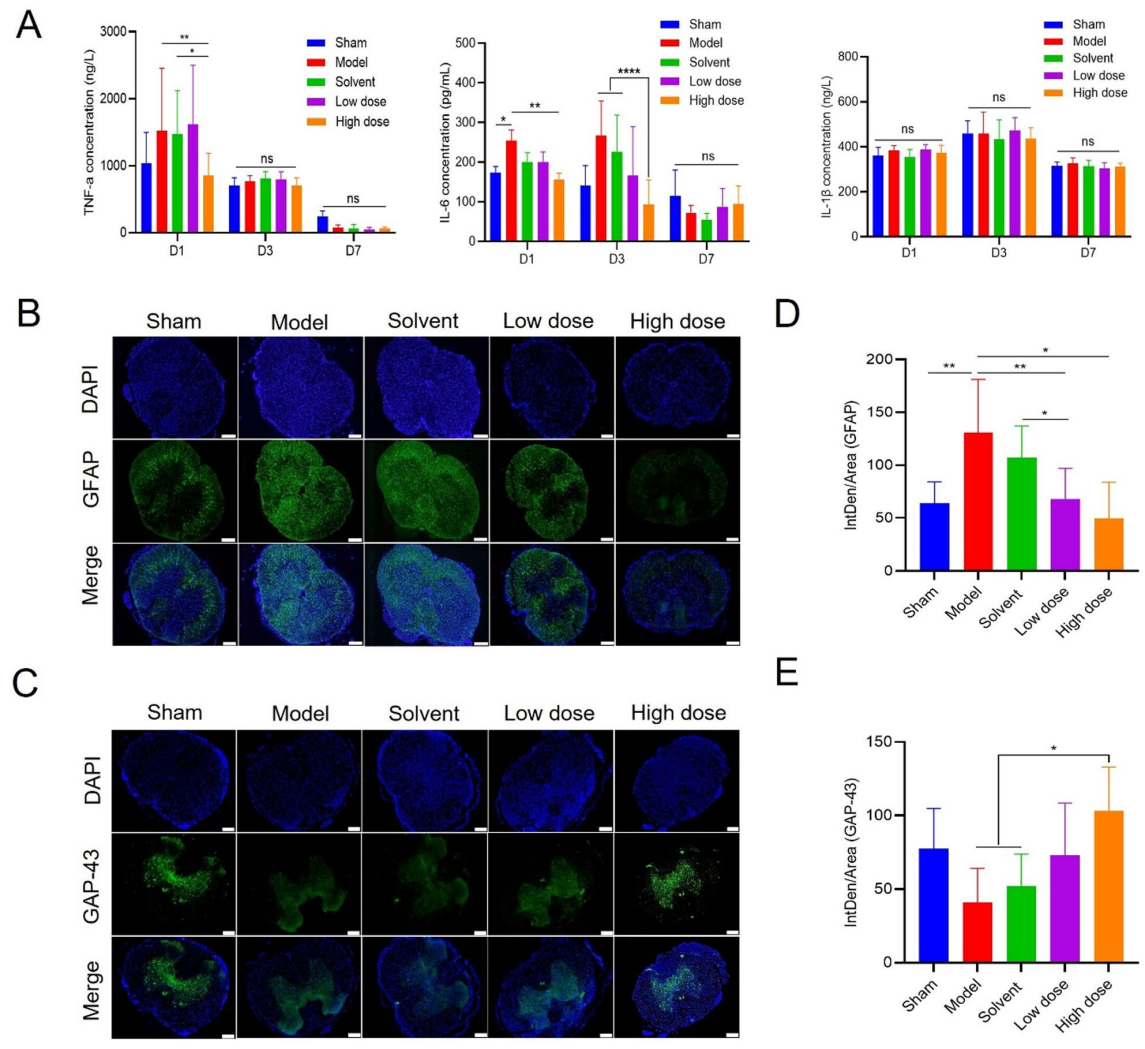
High doses can significantly reduce the inflammatory response and astrogliosis in rats with spinal cord injury. (**A**) ELISA was used to measure the levels of TNF-α, IL-6 and IL-1β in the serum of rats from different treatment groups. (**B**) Immunofluorescence staining was performed to determine the location and expression levels of GFAP in spinal cord tissue sections from different treatment groups (200× magnification). (**C**) Semiquantitative analysis was conducted to measure the average fluorescence intensity of the anti-GFAP antibody in spinal cord sections. (**D**) Immunofluorescence staining was performed to determine the location and expression levels of GAP-43 in spinal cord tissue sections from different treatment groups (200× magnification). (**E**) Semiquantitative analysis was conducted to measure the average fluorescence intensity of the anti-GAP-43 antibody in spinal cord sections. Statistical analysis was performed using one-way analysis of variance (ANOVA) followed by Tukey’s post-hoc test; ^*^P < 0.05, ^**^P < 0.01, ^****^P < 0.0001,* ns* = not significant (*P > 0.05*)

To investigate the modulation of inflammatory responses by UC-MSCs in SCI rats, the serum levels of TNF-α, IL-6, and IL-1β were detected at D1, D3, and 7 days post-administration. As shown in Fig. 4A. For TNF-α, at D1, the model and solvent groups presented notably elevated concentrations (1523.00 ± 933.01 ng/L and 1472.84 ± 651.87 ng/L, respectively) compared with the sham groups. Notably, the high-dose group exhibited a statistically significant reduction in TNF-α levels (857.20 ± 331.46 ng/L) compared with both the model (^*^p < 0.05) and solvent (^**^p < 0.01) groups, with levels comparable to those of the sham group, whereas no significant reduction was observed in the low-dose group at this time point. At D3 and D7, the TNF-α levels gradually decreased across all the groups, with no significant intergroup differences (*p > 0.0*5). Similarly, at 24 h post-administration, the serum IL-6 concentration in the model groups were significantly higher than in the sham group (253.87 ± 27.02 pg/ml vs. 172.84 ± 16.02 pg/ml, ^*^p < 0.05) at 24 h post-administration, while the high-dose group showed a robust, significant reduction in IL-6 to 155.62 ± 16.20 pg/ml (^**^p < 0.01 vs.. model). At 72 h, the IL-6 levels in the model and solvent groups increased further (267.56 ± 86.75 pg/ml and 226.45 ± 92.22 pg/ml, respectively), whereas UC-MSCs treatment induced a downward trend: 165.61 ± 123.87 pg/ml in the low-dose groups and a more significant reduction to 92.71 ± 62.24 pg/ml in the high-dose groups (^****^p < 0.0001). At 7 days, IL-6 concentrations decreased across all groups, with no significant intergroup differences noted (*p > 0.05*). For serum IL-1β, no inter-group differences were detected at any time point, although overall levels tended to decrease at 72 h post-administration. Collectively, these data demonstrate that intravenous administration of high-dose UC-MSCs exerts a targeted, significant suppressive effect on the acute-phase elevation of key pro-inflammatory cytokines TNF-α and IL-6 at the critical early stages of SCI, thereby effectively alleviating the acute inflammatory response.

To investigate astrocyte activation, neuronal plasticity following SCI, and the effects of human umbilical cord mesenchymal stem cells, immunofluorescence staining for glial fibrillary acidic protein (GFAP, an astrocyte marker) and growth-associated protein 43 (GAP-43, a neuronal plasticity marker) was conducted on spinal cord sections. As depicted in Fig. 4B, GFAP was expressed at relatively low levels in the sham group, whereas a marked increase in GFAP fluorescence intensity was observed in the model group, indicating extensive astrocyte activation. Compared with the model group, the solvent treatment slightly reduced GFAP expression; the low-dose groups presented further decreases in GFAP levels, and the high-dose groups presented significant reductions in GFAP expression, with GFAP expression approaching that of the sham group. Quantitative analysis of GFAP fluorescence intensity (Fig. 4C) verified these results: the model group had a significantly greater intensity (^**^p < 0.01) than did the sham group. Solvent treatment led to a slight decrease, and both the low-dose (*p < 0.05) and high-dose (**p < 0.01) groups presented significant reductions, with the high-dose group exerting the most pronounced effect. For GAP-43 (Fig. 4D), the sham group displayed a certain level of GAP-43 fluorescence, whereas the model group presented a notable decrease in GAP-43 expression. The solvent had minimal effects, yet the low-dose and particularly the high-dose groups presented increased GAP-43 fluorescence intensity. Quantitative analysis (Fig. 4E) revealed that the model group presented a significantly lower GAP-43 intensity than the sham group did, and the high-dose groups presented significantly (*p < 0.05) elevated GAP-43 expression, suggesting enhanced neuronal plasticity. Immunohistochemistry (Supplemental Fig. 2) also confirmed these results, with the high-dose group showing significantly smaller GFAP- and GAP-43-positive areas than the other treatment groups did. Collectively, these findings indicate that intravenous administration of high doses significantly reduces the number of GFAP-positive astrocytes, alleviates glial scarring, and improves locomotor function by increasing GAP-43 protein expression.

### Single-cell transcriptomic analysis of PBMCs from SCI rats in the high-dose groups

**Fig. 5:**
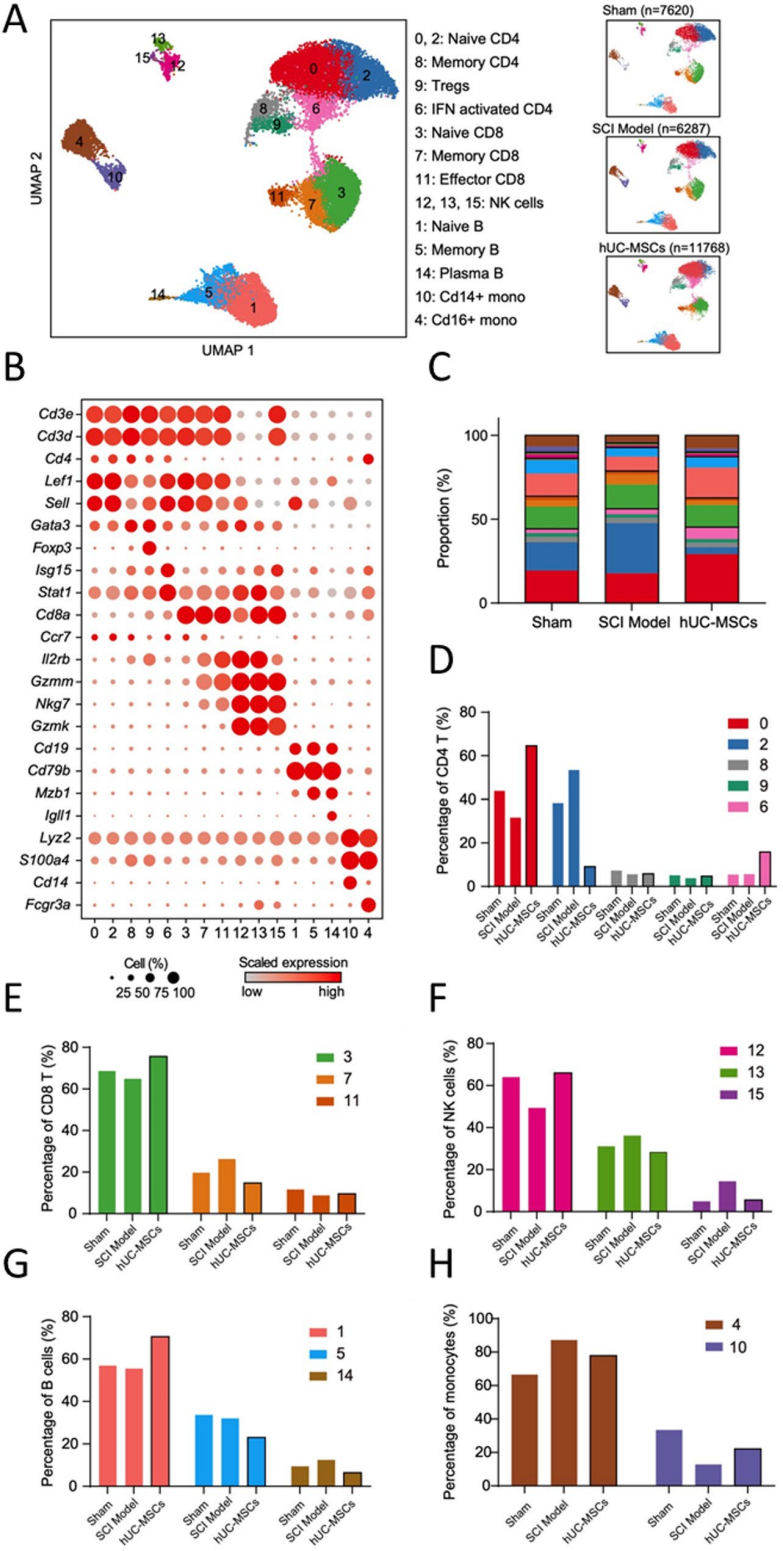
Single-cell transcriptomic analysis of PBMCs from rats subjected to different treatments after SCI. (**A**) UMAP image showing 15 clusters of PBMCs from the sham, model and high-dose groups. PBMCs were projected together by UMAP (left) and displayed separately by experimental group (right). (**B**) Dot plots showing the expression values of representative highly expressed genes for each PBMC cluster. The color represents the scaled expression values from the Seurat RNA assay. (**C**) Stacked bar plot displaying the proportions of various PBMC clusters in the sham group, model group and high-dose groups of rats. Bar plot showing the fractions of CD4^+^ T-cell subpopulations (**D**), CD8^+^ T-cell subpopulations (**E**), NK cell subpopulations (**F**), B-cell subpopulations (**G**) and monocyte subpopulations (**H**). Differential gene expression between groups was performed using the Wilcoxon rank-sum test with Benjamini-Hochberg correction for multiple comparisons (adjusted *p* < 0.05). All scRNA-seq analyses were implemented in the Seurat package (*v4.4.0*) of R software (*v4.3.1*). Differentially expressed genes were defined as |log2 fold change (FC)| >1 and adjusted *p* < 0.05

**Fig. 6:**
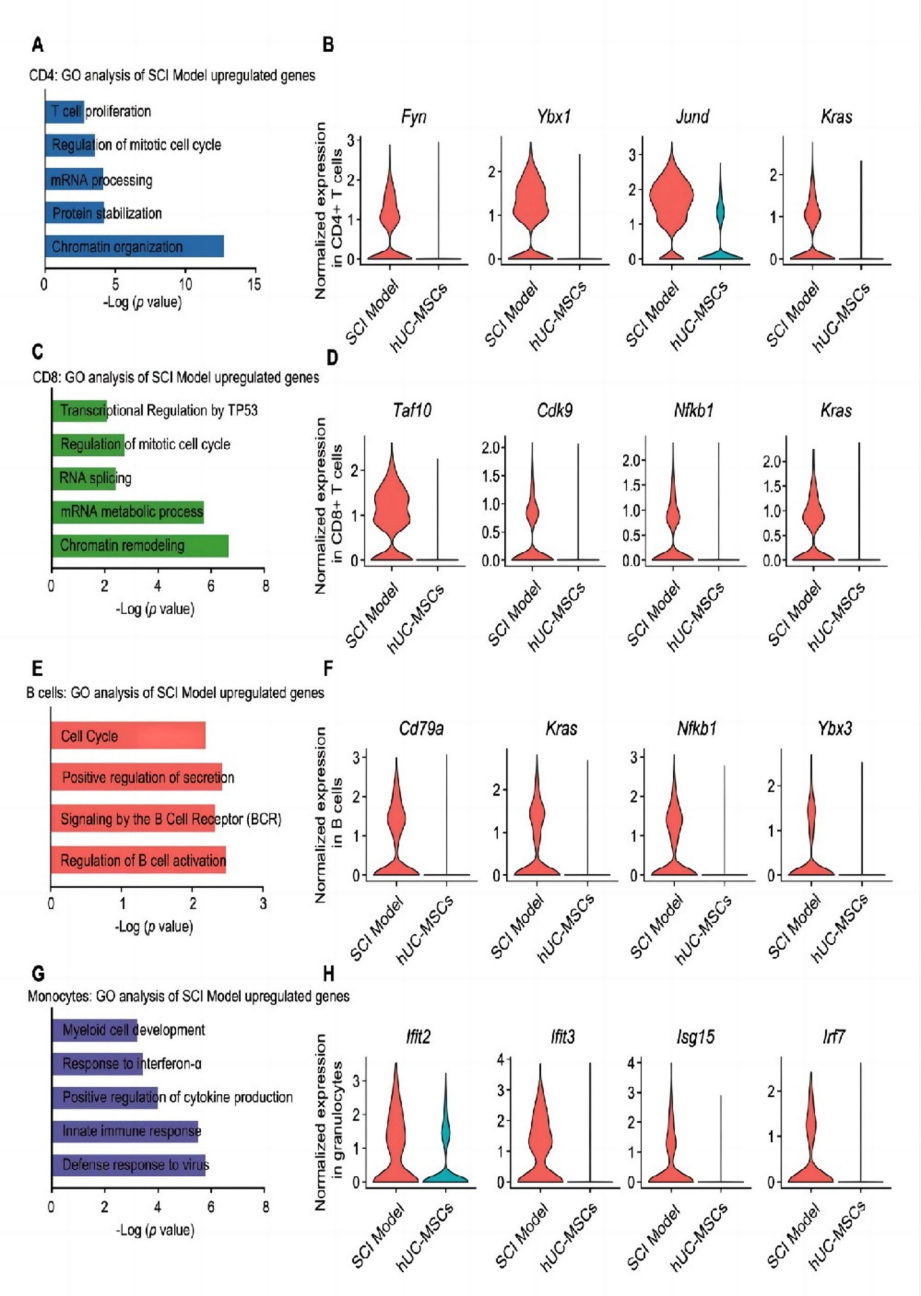
Rats treated with UC-MSCs exhibit modulated immune responses after SCI. (**A**) Bar plots displaying the upregulated Gene Ontology pathways in CD4^+^ T cells from the model groups. (**B**) Violin plots comparing the normalized expression of selected feature genes in CD4 + T cells between the model group and the high-dose group. (**C**) Bar plots displaying the upregulated Gene Ontology pathways in CD8^+^ T cells from the model group. (**D**) Violin plots comparing the normalized expression of selected feature genes in CD8^+^ T cells between the model group and the high-dose group. (**E**) Bar plots displaying the upregulated Gene Ontology pathways in B cells from the model group. (**F**) Violin plots comparing the normalized expression of selected feature genes in B cells from the model group and the high-dose group. (**G**) Bar plots displaying the upregulated Gene Ontology pathways in monocytes from the model group. (**H**) Violin plots comparing the normalized expression of selected feature genes in monocytes from the model group and the high-dose group

To observe the changes in lymphocyte subpopulations in SCI rats after MSC administration and identify the target lymphocytes of UC-MSCs, we collected PBMCs from rats in the sham group, model group, and high-dose group for single-cell transcriptomic analysis, as shown in Fig. 5. UMAP analysis revealed that the PBMCs from the rats in the different treatment groups could be divided into 15 distinct cell clusters (Fig. 5A). As shown in Fig. 5B, Cd3e and Cd3d were highly expressed in CD4 T-cell clusters (0, 2, 8, 9) and CD8 T-cell clusters (3, 7, 11), with Lef1 and Sell additionally enriched in naïve CD4/CD8 T cells (clusters 0, 2, 3). Gata3 was highly expressed in memory CD4 T cells (cluster 8), whereas Foxp3 was coexpressed with Gata3 in regulatory T cells (cluster 9). IFNγ-activated CD4 T cells (cluster 6) presented elevated expression of Lef1, Sell, Isg15, and Stat1. For CD8 + T cells, the effector subset (cluster 11) was characterized by high Cd8a, Il2rb, and Gzmm expression. Natural killer (NK) cell clusters (12, 13, 15) prominently expressed Gzmm, Nkg7, and Gzmk, with cluster 15 also showing Cd3e, Cd3d, and Cd8a expression and clusters 12 and 13 expressing Stat1 and Il2rb. B-cell clusters (1, 5, 14) presented high Cd79b and Cd19 expression, with Mzb1 enriched in memory (cluster 5) and plasma B cells (cluster 14). Monocytes are characterized by Lyz2 and S100a4 expression, with CD14⁺ monocytes expressing Cd14 and CD16⁺ monocytes expressing Cd4 and Fcgr3a. Additionally, the proportions of the 15 cell clusters in PBMCs from the rats in the different treatment groups were presented in Fig. 5C. The variations in the proportions of diverse cell clusters, including CD4⁺ T cells, CD8⁺ T cells, natural killer (NK) cells, B cells, and monocytes, across various treatment groups are depicted in Fig. 5(D-H). Overall, naïve CD4 T lymphocytes (clusters 0 and 2), naïve CD8 T lymphocytes (cluster 3), naïve B lymphocytes (cluster 1), NK cells (cluster 12), and CD16⁺ monocytes (cluster 4) were relatively more abundant in the PBMCs from the rats in all the groups. Compared with those of rats in the model group, the percentage changes in different cell clusters in rats treated with umbilical cord-derived mesenchymal stem cells were as follows: (i) The percentages of naïve CD4 T cells (cluster 0) and interferon-γ (IFNγ)-activated CD4 T cells (cluster 6) increased significantly, whereas the percentage of regulatory T cells (Tregs, cluster 9) slightly increased, and the percentage of naïve CD4⁺ T lymphocytes (cluster 2) tended to decrease (Fig. 5D); (ii) The percentage of naïve CD8 T cells (cluster 3) increased, the percentage of memory CD8 T cells (cluster 7) decreased, and the percentage of effector CD8 T cells (cluster 11) remained unchanged (Fig. 5E); (iii) NK cells displayed three distinct subpopulations, with the proportion of NK cells in cluster 12 increasing, whereas those in clusters 13 and 15 showed opposite trends (Fig. 5F). (iv) The proportion of naïve B cells (cluster 1) increased, whereas the proportions of memory B cells (cluster 5) and plasma B cells (cluster 14) decreased (Fig. 5G). In addition, the proportion of CD16⁺ monocytes (cluster 4) decreased, whereas the proportion of CD14⁺ monocytes (cluster 10) slightly increased (Fig. 5H). Notably, following spinal cord injury (SCI), CD4 T cells, CD8 T cells, B cells, and monocytes in rats significantly upregulated multiple Gene Ontology (GO) pathways, as shown in Fig. 6. In CD4⁺ T cells, genes significantly upregulated post-spinal cord injury (SCI) was enriched in Gene Ontology (GO) pathways, including chromatin organization, protein stabilization, mRNA processing, mitotic cell cycle regulation, and T-cell proliferation (Fig. 6A); among these genes, Fyn, Ybx1, Jund, and Kras, which were markedly upregulated post-SCI, were substantially reduced following UC-MSCs treatment (Fig. 6B). In CD8⁺ T cells, SCI-induced upregulated genes (e.g., Taf10, Cdk9, Nfkb1, and Kras) were associated with GO pathways such as chromatin remodeling, mRNA metabolism, and RNA splicing (Fig. 6C), and these genes were also significantly downregulated after UC-MSCs intervention (Fig. 6D). In B cells, SCI-upregulated genes (e.g., Cd79a, Kras, Nfkb1, and Ybx3) participate in pathways regulating B-cell activation, B-cell receptor (BCR) signaling, secretion positive regulation, and cell cycle progression (Fig. 6E), and these genes were similarly downregulated after UC-MSCs treatment (Fig. 6F). In monocytes, the SCI-induced upregulated genes were involved in signaling pathways, including the viral defense response, the innate immune response, and the positive regulation of cytokine production (Fig. 6G); specifically, the SCI-induced upregulated genes, such as Ifit2, Ifit3, Isg15, and Irf7, were significantly downregulated after UC-MSCs treatment (Fig. 6H). Collectively, these results indicate that UC-MSCs treatment modulates the clustering and proportions of peripheral blood lymphocytes in SCI rats, and by precisely regulating genes and signaling pathways abnormally activated in distinct lymphocyte subsets under SCI stress, UC-MSCs reduce lymphocyte-mediated immune responses, mitigate secondary injury cascades (e.g., inflammation), and prevent further injury exacerbation.

### In vivo dynamics analysis and principal component analysis of UC-MSCs administered after spinal cord injury

**Fig. 7:**
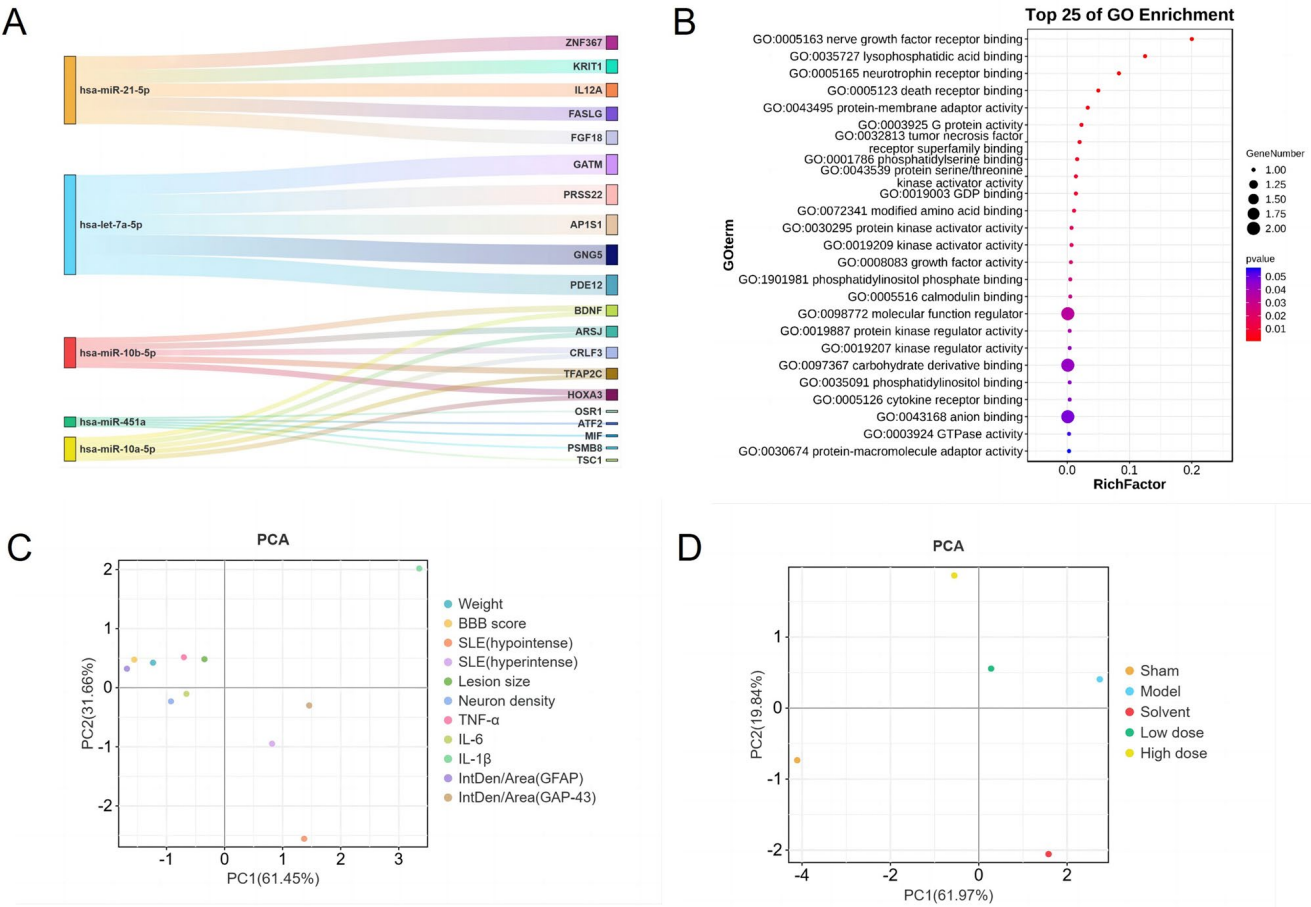
The intravenous administration of UC-MSCs improved SCI through paracrine effects. (**A**) Chord diagram depicting the associations between human-specific sRNAs and their predicted target genes. Each ribbon links an sRNA to its corresponding target gene, illustrating complex regulatory relationships. (**B**) GO analysis of exosomal sRNAs. (**C**) Principal component analysis (PCA) illustrating the correlations among various parameters (body weight, BBB score, SLE hypointense and hyperintense regions, lesion size, neuron density, levels of TNF-α, IL-6, and IL-1β, integrated area density of GFAP, and integrated area density of GAP-43) across different treatment groups. (**D**) Principal component analysis (PCA) plot showing the distributions of different treatment groups (sham, model, solvent, low-dose, and high-dose) on the basis of the aforementioned parameters. Exosomal sRNA sequencing differential analysis was performed using DESeq2 software, with differentially expressed sRNAs defined as |log2 fold change (FC)|>1 and adjusted *p* < 0.05 (Benjamini-Hochberg correction for multiple comparisons). GO enrichment analysis of sRNA target genes was conducted *via* the hypergeometric test with Benjamini-Hochberg correction (adjusted *p* < 0.05 as the significance threshold for pathways). PCA was implemented using XLSTAT software (Addinsoft, New York, USA) and OmicShare tools, with variance explained by principal component 1 (PC1) and principal component 2 (PC2) indicated in parentheses

To enable in vivo tracking of UC-MSCs, we constructed a lentiviral vector encoding the dual reporter genes Fluc and eGFP (pLV-Fluc-eGFP), allowing simultaneous bioluminescence imaging (BLI) and fluorescent detection (Supplemental Fig. 3A). UC-MSCs were transduced with the lentivirus, and flow cytometric analysis confirmed a high and uniform transduction efficiency, with 95.2% of cells positive for IgG1-FITC (Supplemental Fig. 3B). Robust Fluc-derived bioluminescent signals were detected across a series of cell densities in vitro, with signal intensity correlating positively with cell number. These results demonstrate the stable expression and reliable detectability of the dual reporters in UC-MSCs, supporting their use for subsequent in vivo cell-retention and spatiotemporal tracking studies (Supplemental Fig. 3C). In vivo tracking of UC-MSCs products revealed the temporal‒spatial distribution profile. As shown in Supplemental Fig. 4, the left rat served as a negative control, while the right rat was administered UC-MSCs products. At 1 h, 2 h, and 4 h postadministration, obvious luminescence signals were detected in the lung region of the right rat, indicating the initial distribution of the administered products. By 8 h, the luminescence signals started to diminish and became more dispersed. On day 1, only faint signals were observable, and by day 3, the luminescence was barely detectable, suggesting that the UC-MSCs products were gradually metabolized and cleared from the body over time. Furthermore, as shown in Fig. 7A, our findings revealed a distinct panel of human-specific sRNAs that exhibited significant alterations in abundance postinfusion, with several key species showing marked upregulation, such as hsa-miR-21-5p, hsa-let-7a-5p, hsa-miR-10b-5p, hsa-miR-451a, and hsa-miR-10a-5p. Moreover, hsa-miR-21-5p was predicted to target genes such as *ZNF367*, *KRIT1*, *IL12A*, *FASLG*, and *FGF18*; hsa-let-7a-5p was associated with *GATM*, *PRSS22*, *AP1S1*, *GNG5*, and *PDE12*; hsa-miR-10b-5p potentially targeted *BDNF*, *ARSJ*, *CRLF3*, *TFAP2C*, and *HOXA3*; hsa-miR-451a was linked to *OSR1*, *ATF2*, *MIF*, *PSMB8*, and *TSC1*; and hsa-miR-10a-5p also exhibited predicted interactions with these aforementioned target genes. sRNA sequencing analysis was performed to dissect the molecular cargo of human-derived exosomes, with a chord diagram illustrating the intricate associations between distinct sRNAs and their potential target elements, reflecting the complexity of human exosomal sRNA-mediated regulatory networks. Furthermore, Gene Ontology (GO) enrichment analysis focusing on the top 25 terms (Fig. 7B) revealed that these sRNAs were involved primarily in functional processes, including growth factor receptor binding, transferase activity, and receptor binding, which are pivotal for regulating cell proliferation, differentiation, and intercellular communication. Collectively, these findings delineate the in vivo behavior of UC-MSCs products and the molecular underpinnings of sRNA-mediated regulation, providing a foundation for understanding their therapeutic mechanisms. To further elucidate the comprehensive effects of UC-MSCs on spinal cord injury (SCI), principal component analysis (PCA) was performed, as shown in the supplemental results of Table S1. In Fig. 7C, the PCA plot illustrates the relationships among various parameters, including weight, Basso–Beattie–Bresnahan (BBB) score, signal alterations on spinal lesion evaluation (SLE, hypointense and hyperintense), lesion size, neuron density, proinflammatory cytokines (TNF-α, IL-6, and IL-1β), and the intensity density per area of GFAP (IntDen/Area (GFAP)) and GAP-43 (IntDen/Area (GAP-43)). PC1 accounted for 61.45% of the variance, and PC2 accounted for 31.66%, revealing distinct clustering and correlations that reflected the multifaceted impacts of SCI and potential therapeutic responses. Moreover, Fig. 7D shows the PCA distributions of the different groups (sham, model, solvent, low-dose, and high-dose groups). Here, PC1 explained 61.97% of the variance, and PC2 explained 19.84%, indicating clear separation among the groups. Compared with the other groups, the high-dose group exhibited a unique clustering pattern, suggesting that it induced a distinct profile in terms of the integrated parameters, which might be associated with its superior therapeutic efficacy in modulating SCI-related pathological and functional changes.

## Discussion

**Fig. 8:**
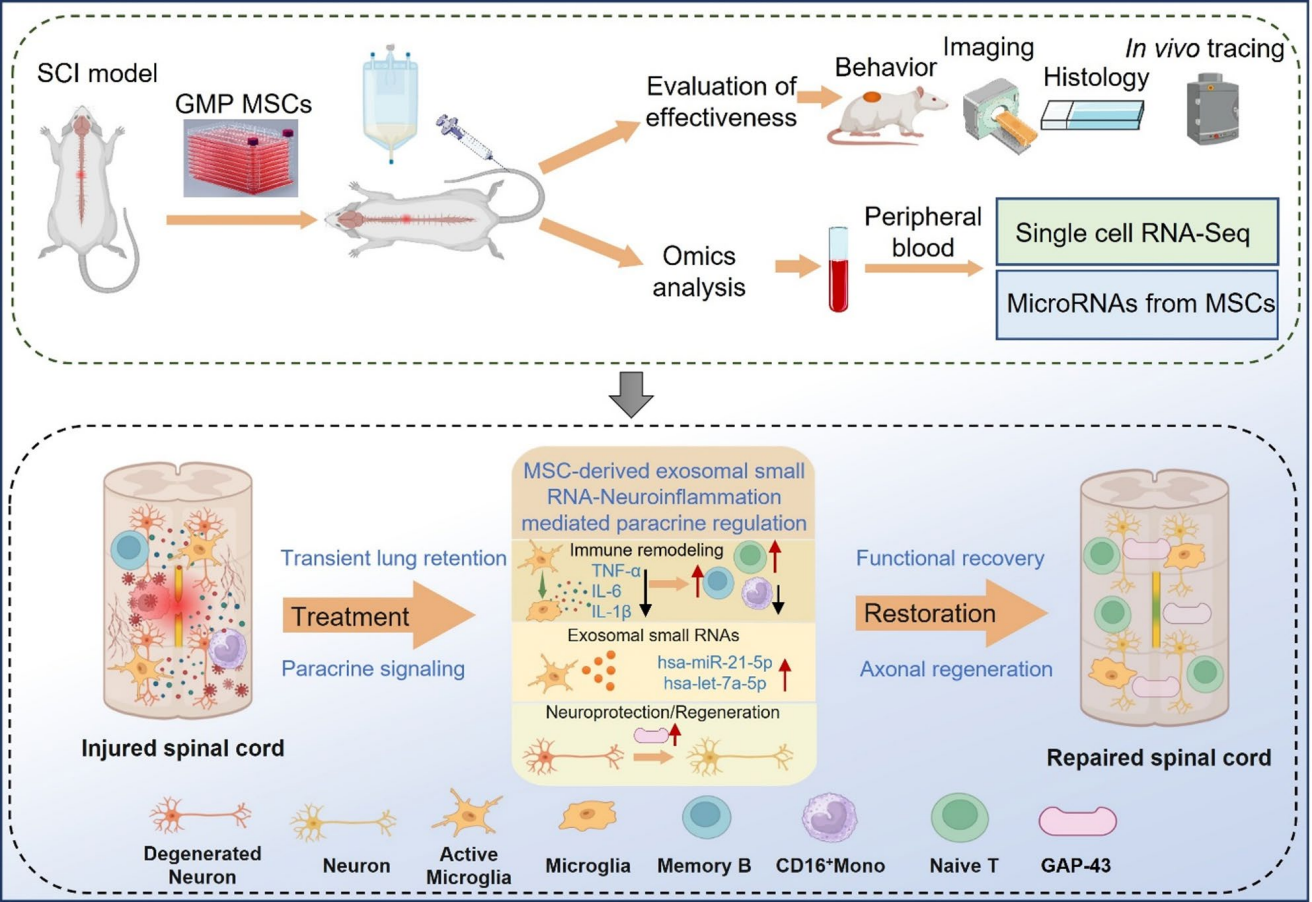
Schematic diagram of the molecular mechanism by which UC-MSCs mediate injured spinal cord repair. Schematic illustration depicts the delivery of human-specific small RNAs (sRNAs) *via* UC-MSC-derived exosomes into the injured spinal cord microenvironment, coupled with their multidimensional regulatory functions through key gene targeting. This therapeutic paradigm orchestrates three interwoven core reparative processes: (1) immune remodeling, UC-MSCs suppress the release of proinflammatory cytokines (TNF-α, IL-1β, and IL-6) to mitigate excessive neuroinflammation, while modulating the activity of immune cells (CD4⁺T, CD8⁺T, NK, B cells, and monocytes), thereby balancing immune responses to prevent pathological immune attacks on residual neural tissue and creating a favorable microenvironment for tissue repair; (2) exosomal small RNAs, UC-MSCs release paracrine factors (notably hsa-miR-21-5p and hsa-let-7a-5p), which mediates intercellular crosstalk within the injured spinal cord microenvironment; and (3) neuroprotection/regeneration, as the expression of growth-associated protein 43 (GAP-43), a critical marker for axonal sprouting and neuronal regeneration, facilitates the reconstruction of damaged neural circuits

A core finding of this study is that intravenous administration of a high-doses GMP-grade human UC-MSCs (3 × 10⁷ cells/kg) drives robust functional and structural recovery in SCI rats, outperforming the low-dose groups (1 × 10⁷ cells/kg), solvent, and model groups. Behavioral assessments *via* the Basso-Beattie-Bresnahan (BBB) scale demonstrated that high-dose groups significantly improved hindlimb locomotor function from post-treatment day 7 to day 21, with scores approaching near-normal levels by the study endpoint. This functional recovery was corroborated by structural evidence: gross morphology and MRI revealed that the high-dose groups presented reduced spinal cord cavity formation and prevented the exacerbation of edema/bleeding, whereas the model and solvent groups presented severe tissue disruption—including near-complete severance of the spinal cord bundle. Histological analyses further confirmed that the high-dose groups exhibited preserved neuronal morphology, increased neuron density, and reduced infiltration of inflammatory cells. Notably, the solvent group showed no significant improvements relative to the model group, ruling out nonspecific effects of the injection vehicle and confirming that UC-MSCs are the driver of therapeutic benefit (Fig. 8). These results highlight the neuroprotective potential of MSCs in SCI, but extend this work by validating that GMP-grade cells are critical for ensuring clinical safety, purity, and standardization. While prior preclinical studies have confirmed the therapeutic potential of MSCs for SCI, most have relied on non-GMP-grade cell products or local delivery routes (intrathecal/intraspinal) with limited clinical translatability; our study is the first to validate the efficacy of GMP-grade human UC-MSCs *via* intravenous administration, a route that is far more feasible for clinical application.

Inflammation is a central mediator of secondary SCI injury, with excessive proinflammatory cytokines (e.g., TNF-α and IL-6) amplifying neuronal loss and glial scarring. Our ELISA data revealed that the high-dose GMP-grade UC-MSCs exert a targeted, time-dependent suppression of acute-phase inflammation: they had reduced serum TNF-α levels at 24 h post-administration and IL-6 levels at both 24 h and 72 h, whereas the low-dose group had minimal effects on these cytokines. IL-1β levels showed no inter-group differences, likely because of its earlier peak in the inflammatory cascade or its lower sensitivity to UC-MSC-mediated modulation. This anti-inflammatory effect was further supported by the immunofluorescence results: the high-dose groups presented reduced expression of glial fibrillary acidic protein (GFAP), a marker of astrocyte activation and glial scarring [30, 31]. Glial scars are major barriers to axonal regeneration, and their attenuation by UC-MSCs likely creates a permissive microenvironment for neuronal repair [32, 33]. Conversely, high-dose groups presented increased expression of growth-associated protein 43 (GAP-43), a key marker of neuronal plasticity and axonal sprouting [34–37]. Together, these findings confirm that immunomodulation, coupled with the promotion of neuroprotective and regenerative factors, is a central mechanism of UC-MSCs action in SCI, which is consistent with reports that MSCs secrete anti-inflammatory molecules and neurotrophic factors to dampen excessive inflammation and support neuronal survival [38–40]. What distinguishes our findings from prior work is the demonstration of this mechanism in the context of clinically approved GMP-grade UC-MSCs and intravenous delivery, and the identification of a clear dose-dependent effect on acute inflammatory suppression—an essential detail for guiding subsequent clinical trial design.

To elucidate the immunomodulatory mechanisms of UC-MSCs, we performed single-cell RNA sequencing (scRNA-seq) on peripheral blood mononuclear cells (PBMCs) from the sham, model, and high-dose groups. UMAP analysis revealed 15 distinct immune cell clusters, and proportional changes revealed that UC-MSCs reshaped the SCI-induced immune landscape: they increased the proportions of naïve CD4⁺/CD8⁺ T cells and naïve B cells; reduced the proportions of memory B cells, plasma cells, and CD16⁺ monocytes; and redistributed natural killer (NK) cell subpopulations [41]. This shift from a proinflammatory effector state to a quiescent, regulatory state aligns with our serological and histological data showing reduced cytokine levels and inflammatory infiltration. GO pathway analysis further revealed that SCI upregulated genes involved in T-cell proliferation (e.g., Jund, Fyn), B-cell activation (e.g., Nfkb1), and innate immune responses (e.g., Kras, Isg15) in lymphocytes and monocytes; high-dose UC-MSCs downregulated these genes. Notably, Jund (an AP-1 family transcription factor) regulates T-cell cytokine secretion [42, 43], Fyn (a Src family kinase) mediates T-cell receptor signaling [44, 45], Nfkb1 (an NF-κB subunit) drives inflammatory gene expression [46, 47], and Kras modulates T-cell function and immune evasion [48–50]; their suppression likely contributes to attenuated immune activation and secondary injury. These transcriptomic insights highlight the ability of UC-MSCs to target both adaptive and innate immunity—a nuanced mechanism not fully captured in prior studies.

In vivo tracking via bioluminescence imaging (BLI) revealed that UC-MSCs initially accumulated in the lungs (1–4 h post-administration) and were cleared by day 3, which is consistent with the pulmonary first-pass effect of intravenously delivered MSCs. This transient presence suggests that their therapeutic effects are mediated primarily through paracrine mechanisms rather than long-term engraftment, a hypothesis supported by our exosome sRNA sequencing data. We also identified human-specific miRNAs (hsa-miR-21-5p, hsa-let-7a-5p, hsa-miR-10b-5p, hsa-miR-451a, and hsa-miR-10a-5p) in rat serum after UC-MSC administration, which supports the growing consensus that MSC-derived exosomes *via* their sRNA cargo are major mediators of therapeutic effects, suggesting a potential cell-free alternative for SCI treatment.

A key objective of this study was to define an optimal UC-MSCs dosage for SCI, and our results clearly demonstrated dose-dependent efficacy: the high dose (3 × 10⁷ cells/kg) outperformed the low dose across all functional, structural, and molecular endpoints. This is critical for clinical translation, as it provides a starting point for human dose–escalation trials. Additionally, the use of GMP-grade UC-MSCs ensures that our findings are clinically relevant, as GMP compliance is mandatory for cell-based therapies to guarantee safety and consistency.

This study has inherent limitations that guide future research priorities. Findings are based on a rat T10 contusion SCI model, and validation in larger animal models is needed to better recapitulate human SCI pathology. The 21-day follow-up only reflects short-term therapeutic effects; long-term evaluations (3–6 months) are required to confirm the sustainability of UC-MSC-mediated recovery and the long-term safety of high-dose administration. While human-derived exosomal miRNAs were identified as paracrine mediators, causal validation *via* exosome depletion or miRNA manipulation remains unperformed, this is critical for optimizing cell-free therapeutic strategies. Despite these limitations, our study delivers novel translational contributions to human UC-MSCs-based SCI therapy: we are the first to demonstrate the dose-dependent efficacy of NMPA-approved GMP-grade human UC-MSCs *via* intravenous delivery, identify a clinically relevant optimal dosage, uncover high-resolution integrated immunomodulatory mechanisms through scRNA-seq, and characterize GMP-grade UC-MSC-derived exosomal miRNAs that mediate paracrine effects.

## Conclusion

This study demonstrated that GMP-grade UC-MSCs exert dose-dependent therapeutic effects on traumatic SCI in rats, with a high dose (3 × 10⁷ cells/kg) being the optimal regimen. These benefits are mediated through multiple mechanisms: mitigating acute inflammation, suppressing glial scarring, promoting neuronal plasticity and survival, reshaping the peripheral immune landscape, and delivering exosomal miRNAs. These findings provide critical preclinical evidence for the clinical translation of GMP-grade UC-MSCs in SCI and highlight the need for further research to address remaining translational barriers.

## Supplementary Information

Below is the link to the electronic supplementary material.

Supplementary Material 1

## Data Availability

All results and data in our study are available from the corresponding author upon reasonable request.

## Abbreviations

AD: Alzheimer’s disease
BBB: Basso-Beattie-Bresnahan
BLI: Bioluminescence imaging
CNS: Central nervous system
GAP-43: Growth-associated protein 43
GFAP: Glial fibrillary acidic protein
GMP: Good manufacturing practice
GO: Gene Ontology
H&E: Hematoxylin and eosin
IFN-γ: Interferon gamma
IGF-1: Insulin-like growth factor 1
IL-1β: Interleukin-1 beta
IL-6: Interleukin-6
MFI: Mean fluorescence intensity
MRI: Magnetic resonance imaging
NMPA: National Medical Products Administration
PCA: Principal component analysis
PPI: Protein-protein interaction
SCI: Spinal cord injury
SILE: Lesion signal intensity
scRNA-seq: Single-cell RNA sequencing
sRNA: Small RNA
BLI: Bioluminescence imaging
TNF-α: Tumor necrosis factor-alpha
Treg: Regulatory T cell
UC-MSCs: Umbilical cord-derived mesenchymal stem cells

## References

[1] Ahuja CS, Wilson JR, Nori S, Kotter MRN, Druschel C, Curt A, et al. Traumatic spinal cord injury. Nat Rev Dis Primers. 2017;3:17018. 10.1038/nrdp.2017.18. 28447605 10.1038/nrdp.2017.18

[2] Yuan T, Li W, Zhou M, Wang X, Wang B, Zhao Y. Biomimetic Multichannel Silk Nerve Conduits With Multicellular Spatiotemporal Distributions for Spinal Cord Injury Repair. Adv Mater. 2024;36(44):e2411628. 10.1002/adma.202411628. 39268784 10.1002/adma.202411628

[3] Jiang B, Sun D, Sun H, Ru X, Liu H, Ge S, et al. Prevalence, Incidence, and External Causes of Traumatic Spinal Cord Injury in China: A Nationally Representative Cross-Sectional Survey. Front Neurol. 2021;12:784647. 10.3389/fneur.2021.784647. 35126291 10.3389/fneur.2021.784647 PMC8811043

[4] Morishima Y, Kawabori M, Yamazaki K, Takamiya S, Yamaguchi S, Nakahara Y, et al. Intravenous administration of mesenchymal stem cell-derived exosome alleviates spinal cord injury by regulating neutrophil extracellular trap formation through exosomal miR-125a-3p. Int J Mol Sci. 2024;25(4). 10.3390/ijms25042406. 10.3390/ijms25042406 PMC10889446 38397083

[5] Punjani N, Deska-Gauthier D, Hachem LD, Abramian M, Fehlings MG. Neuroplasticity and regeneration after spinal cord injury. N Am Spine Soc J. 2023;15:100235. 10.1016/j.xnsj.2023.100235. 37416090 10.1016/j.xnsj.2023.100235 PMC10320621

[6] Yang L, Cao J, Du Y, Zhang X, Hong W, Peng B, et al. Initial IL-10 production dominates the therapy of mesenchymal stem cell scaffold in spinal cord injury. Theranostics. 2024;14(2):879–91. 10.7150/thno.87843. 38169599 10.7150/thno.87843 PMC10758068

[7] Squair JW, Milano M, de Coucy A, Gautier M, Skinnider MA, James ND, et al. Recovery of walking after paralysis by regenerating characterized neurons to their natural target region. Science. 2023;381(6664):1338–45. 10.1126/science.adi6412. 37733871 10.1126/science.adi6412

[8] Zipser CM, Cragg JJ, Guest JD, Fehlings MG, Jutzeler CR, Anderson AJ, et al. Cell-based and stem-cell-based treatments for spinal cord injury: evidence from clinical trials. Lancet Neurol. 2022;21(7):659–70. 10.1016/S1474-4422(21)00464-6. 35569486 10.1016/S1474-4422(21)00464-6

[9] Dell’Anno MT, Strittmatter SM. Rewiring the spinal cord: Direct and indirect strategies. Neurosci Lett. 2017;652:25–34. 10.1016/j.neulet.2016.12.002. 28007647 10.1016/j.neulet.2016.12.002 PMC5466898

[10] Zhou J, Shi Y. Mesenchymal stem/stromal cells (MSCs): origin, immune regulation, and clinical applications. Cell Mol Immunol. 2023;20(6):555–7. 10.1038/s41423-023-01034-9. 37225837 10.1038/s41423-023-01034-9 PMC10229593

[11] Takahashi A, Nakajima H, Kubota A, Watanabe S, Matsumine A. Adipose-derived mesenchymal stromal cell transplantation for severe spinal cord injury: functional improvement supported by angiogenesis and neuroprotection. Cells. 2023;12(11). 10.3390/cells12111470. 10.3390/cells12111470 PMC10252677 37296591

[12] An J, Chen B, Zhang R, Tian D, Shi K, Zhang L, et al. Therapeutic Potential of Mesenchymal Stem Cell-Derived Exosomes in Spinal Cord Injury. Mol Neurobiol. 2024. 10.1007/s12035-024-04490-0. 39312070 10.1007/s12035-024-04490-0

[13] Liang Z, Yang Z, Xie H, Rao J, Xu X, Lin Y, et al. Small extracellular vesicles from hypoxia-preconditioned bone marrow mesenchymal stem cells attenuate spinal cord injury via miR-146a-5p-mediated regulation of macrophage polarization. Neural Regen Res. 2024;19(10):2259–69. 10.4103/1673-5374.391194. 38488560 10.4103/1673-5374.391194 PMC11034578

[14] Pittenger MF, Discher DE, Peault BM, Phinney DG, Hare JM, Caplan AI. Mesenchymal stem cell perspective: cell biology to clinical progress. NPJ Regen Med. 2019;4:22. 10.1038/s41536-019-0083-6. 31815001 10.1038/s41536-019-0083-6 PMC6889290

[15] Liu J, Qi L, Bao S, Yan F, Chen J, Yu S, et al. The acute spinal cord injury microenvironment and its impact on the homing of mesenchymal stem cells. Exp Neurol. 2024;373:114682. 10.1016/j.expneurol.2024.114682. 38199509 10.1016/j.expneurol.2024.114682

[16] Crigler L, Robey RC, Asawachaicharn A, Gaupp D, Phinney DG. Human mesenchymal stem cell subpopulations express a variety of neuro-regulatory molecules and promote neuronal cell survival and neuritogenesis. Exp Neurol. 2006;198(1):54–64. 10.1016/j.expneurol.2005.10.029. 16336965 10.1016/j.expneurol.2005.10.029

[17] Gavasso S, Krakenes T, Olsen H, Evjenth EC, Ytterdal M, Haugsoen JB, et al. The therapeutic mechanisms of mesenchymal stem cells in MS—A review focusing on neuroprotective properties. Int J Mol Sci. 2024;25(3). 10.3390/ijms25031365. 10.3390/ijms25031365 PMC10855165 38338644

[18] Liu Y, Zhao C, Zhang R, Pang Y, Li L, Feng S. Progression of mesenchymal stem cell regulation on imbalanced microenvironment after spinal cord injury. Stem Cell Res Ther. 2024;15(1):343. 10.1186/s13287-024-03914-x. 39354635 10.1186/s13287-024-03914-x PMC11446099

[19] Urrutia DN, Caviedes P, Mardones R, Minguell JJ, Vega-Letter AM, Jofre CM. Comparative study of the neural differentiation capacity of mesenchymal stromal cells from different tissue sources: An approach for their use in neural regeneration therapies. PLoS ONE. 2019;14(3):e0213032. 10.1371/journal.pone.0213032. 30856179 10.1371/journal.pone.0213032 PMC6437714

[20] Wei H, Xu Y, Chen Q, Chen H, Zhu X, Li Y. Mesenchymal stem cell-derived exosomal miR-223 regulates neuronal cell apoptosis. Cell Death Dis. 2020;11(4):290. 10.1038/s41419-020-2490-4. 32341353 10.1038/s41419-020-2490-4 PMC7184756

[21] Giovannelli L, Bari E, Jommi C, Tartara F, Armocida D, Garbossa D, et al. Mesenchymal stem cell secretome and extracellular vesicles for neurodegenerative diseases: Risk-benefit profile and next steps for the market access. Bioact Mater. 2023;29:16–35. 10.1016/j.bioactmat.2023.06.013. 37456581 10.1016/j.bioactmat.2023.06.013 PMC10338239

[22] Hwang J, Jang S, Kim C, Lee S, Jeong HS. Role of stem cell-derived exosomes and microRNAs in spinal cord injury. Int J Mol Sci. 2023;24(18). 10.3390/ijms241813849. 10.3390/ijms241813849 PMC10530823 37762150

[23] Mukkala AN, Jerkic M, Khan Z, Szaszi K, Kapus A, Rotstein O. Therapeutic effects of mesenchymal stromal cells require mitochondrial transfer and quality control. Int J Mol Sci. 2023;24(21). 10.3390/ijms242115788. 10.3390/ijms242115788 PMC10647450 37958771

[24] Bydon M, Qu W, Moinuddin FM, Hunt CL, Garlanger KL, Reeves RK, et al. Intrathecal delivery of adipose-derived mesenchymal stem cells in traumatic spinal cord injury: Phase I trial. Nat Commun. 2024;15(1):2201. 10.1038/s41467-024-46259-y. 38561341 10.1038/s41467-024-46259-y PMC10984970

[25] Zhang X, Kuang Q, Xu J, Lin Q, Chi H, Yu D. MSC-based cell therapy in neurological diseases: a concise review of the literature in pre-clinical and clinical research. Biomolecules. 2024;14(5). 10.3390/biom14050538. 10.3390/biom14050538 PMC11117494 38785945

[26] Hu X, Xu W, Ren Y, Wang Z, He X, Huang R, et al. Spinal cord injury: molecular mechanisms and therapeutic interventions. Signal Transduct Target Ther. 2023;8(1):245. 10.1038/s41392-023-01477-6. 37357239 10.1038/s41392-023-01477-6 PMC10291001

[27] Li S, Zhou J, Zhang J, Wang D, Ma J. Construction of rat spinal cord injury model based on Allen’s animal model. Saudi J Biol Sci. 2019;26(8):2122–6. 10.1016/j.sjbs.2019.09.033. 31889806 10.1016/j.sjbs.2019.09.033 PMC6923460

[28] Martinez M, Brezun JM, Bonnier L, Xerri C. A new rating scale for open-field evaluation of behavioral recovery after cervical spinal cord injury in rats. J Neurotrauma. 2009;26(7):1043–53. 10.1089/neu.2008.0717. 19594382 10.1089/neu.2008.0717

[29] Song RB, Basso DM, da Costa RC, Fisher LC, Mo X, Moore SA. Adaptation of the Basso-Beattie-Bresnahan locomotor rating scale for use in a clinical model of spinal cord injury in dogs. J Neurosci Methods. 2016;268:117–24. 10.1016/j.jneumeth.2016.04.023. 27155106 10.1016/j.jneumeth.2016.04.023 PMC4903932

[30] Middeldorp J, Hol EM. GFAP in health and disease. Prog Neurobiol. 2011;93(3):421–43. 10.1016/j.pneurobio.2011.01.005. 21219963 10.1016/j.pneurobio.2011.01.005

[31] Zheng X, Yang J, Hou Y, Shi X, Liu K. Prediction of clinical progression in nervous system diseases: plasma glial fibrillary acidic protein (GFAP). Eur J Med Res. 2024;29(1):51. 10.1186/s40001-023-01631-4. 38216970 10.1186/s40001-023-01631-4 PMC10785482

[32] Zhu X, Wang Z, Sun YE, Liu Y, Wu Z, Ma B, et al. Neuroprotective effects of human umbilical cord-derived mesenchymal stem cells from different donors on spinal cord injury in mice. Front Cell Neurosci. 2021;15:768711. 10.3389/fncel.2021.768711. 35087378 10.3389/fncel.2021.768711 PMC8787356

[33] Cui L, Luo W, Jiang W, Li H, Xu J, Liu X, et al. Human umbilical cord mesenchymal stem cell-derived exosomes promote neurological function recovery in rat after traumatic brain injury by inhibiting the activation of microglia and astrocyte. Regen Ther. 2022;21:282–7. 10.1016/j.reth.2022.07.005. 36092501 10.1016/j.reth.2022.07.005 PMC9440059

[34] Strittmatter SM, Vartanian T, Fishman MC. GAP-43 as a plasticity protein in neuronal form and repair. J Neurobiol. 1992;23(5):507–20. 10.1002/neu.480230506. 1431834 10.1002/neu.480230506

[35] Chung D, Shum A, Caraveo G. GAP-43 and BASP1 in Axon Regeneration: Implications for the Treatment of Neurodegenerative Diseases. Front Cell Dev Biol. 2020;8:567537. 10.3389/fcell.2020.567537. 33015061 10.3389/fcell.2020.567537 PMC7494789

[36] Frey D, Laux T, Xu L, Schneider C, Caroni P. Shared and unique roles of CAP23 and GAP43 in actin regulation, neurite outgrowth, and anatomical plasticity. J Cell Biol. 2000;149(7):1443–54. 10.1083/jcb.149.7.1443. 10871284 10.1083/jcb.149.7.1443 PMC2175140

[37] Yuan Q, Hu B, Su H, So KF, Lin Z, Wu W. GAP-43 expression correlates with spinal motoneuron regeneration following root avulsion. J Brachial Plex Peripher Nerve Inj. 2009;4:18. 10.1186/1749-7221-4-18. 19852861 10.1186/1749-7221-4-18 PMC2771005

[38] Staff NP, Jones DT, Singer W. Mesenchymal Stromal Cell Therapies for Neurodegenerative Diseases. Mayo Clin Proc. 2019;94(5):892–905. 10.1016/j.mayocp.2019.01.001. 31054608 10.1016/j.mayocp.2019.01.001 PMC6643282

[39] Mattei V, Delle Monache S. Mesenchymal stem cells and their role in neurodegenerative diseases. Cells. 2024;13(9). 10.3390/cells13090779. 10.3390/cells13090779 PMC11083223 38727315

[40] Song N, Scholtemeijer M, Shah K. Mesenchymal Stem Cell Immunomodulation: Mechanisms and Therapeutic Potential. Trends Pharmacol Sci. 2020;41(9):653–64. 10.1016/j.tips.2020.06.009. 32709406 10.1016/j.tips.2020.06.009 PMC7751844

[41] Tatsumi N, Kumamoto Y. Role of mouse dendritic cell subsets in priming naive CD4 T cells. Curr Opin Immunol. 2023;83:102352. 10.1016/j.coi.2023.102352. 37276821 10.1016/j.coi.2023.102352 PMC10524374

[42] Hernandez JM, Floyd DH, Weilbaecher KN, Green PL, Boris-Lawrie K. Multiple facets of junD gene expression are atypical among AP-1 family members. Oncogene. 2008;27(35):4757–67. 10.1038/onc.2008.120. 18427548 10.1038/onc.2008.120 PMC2726657

[43] Meixner A, Karreth F, Kenner L, Wagner EF. JunD regulates lymphocyte proliferation and T helper cell cytokine expression. EMBO J. 2004;23(6):1325–35. 10.1038/sj.emboj.7600133. 15029240 10.1038/sj.emboj.7600133 PMC381408

[44] Cooke MP, Abraham KM, Forbush KA, Perlmutter RM. Regulation of T cell receptor signaling by a src family protein-tyrosine kinase (p59fyn). Cell. 1991;65(2):281–91. 10.1016/0092-8674(91)90162-r. 2015626 10.1016/0092-8674(91)90162-r

[45] Lowin-Kropf B, Kunz B, Schneider P, Held W. A role for the src family kinase Fyn in NK cell activation and the formation of the repertoire of Ly49 receptors. Eur J Immunol. 2002;32(3):773–82. 10.1002/1521-4141(200203)32:3&lt;773::AID-IMMU773&gt;3.0.CO;2-U. 11870621 10.1002/1521-4141(200203)32:3<773::AID-IMMU773>3.0.CO;2-U

[46] Msweli S, Pakala SB, Syed K. NF-kappaB transcription factors: their distribution, family expansion, structural conservation, and evolution in animals. Int J Mol Sci. 2024;25(18). 10.3390/ijms25189793. 10.3390/ijms25189793 PMC11432056 39337282

[47] Guo Q, Jin Y, Chen X, Ye X, Shen X, Lin M, et al. NF-kappaB in biology and targeted therapy: new insights and translational implications. Signal Transduct Target Ther. 2024;9(1):53. 10.1038/s41392-024-01757-9. 38433280 10.1038/s41392-024-01757-9 PMC10910037

[48] Ash LJ, Busia-Bourdain O, Okpattah D, Kamel A, Liberchuk A, Wolfe AL. KRAS: Biology, Inhibition, and Mechanisms of Inhibitor Resistance. Curr Oncol. 2024;31(4):2024–46. 10.3390/curroncol31040150. 38668053 10.3390/curroncol31040150 PMC11049385

[49] Zdanov S, Mandapathil M, Abu Eid R, Adamson-Fadeyi S, Wilson W, Qian J, et al. Mutant KRAS Conversion of Conventional T Cells into Regulatory T Cells. Cancer Immunol Res. 2016;4(4):354–65. 10.1158/2326-6066.CIR-15-0241. 26880715 10.1158/2326-6066.CIR-15-0241 PMC4884020

[50] Chen N, Fang W, Lin Z, Peng P, Wang J, Zhan J, et al. KRAS mutation-induced upregulation of PD-L1 mediates immune escape in human lung adenocarcinoma. Cancer Immunol Immunother. 2017;66(9):1175–87. 10.1007/s00262-017-2005-z. 28451792 10.1007/s00262-017-2005-z PMC5579171

