# Supplementary material for: Translational potential of GMP-grade human umbilical cord-derived mesenchymal stem cells (UC-MSCs) in traumatic spinal cord injury: a preclinical study in rat
Source: J Transl Med. 2026 Jun 10;24:771. doi: 10.1186/s12967-026-08373-x (PMC13270768; doi:10.1186/s12967-026-08373-x)
Supplement: Supplementary file 1 — Supplementary Material 1 [file 12967_2026_8373_MOESM1_ESM.docx]

**Supplemental data**

**Supplemental methods**

**Weight measurement**

Measurements were conducted at the same time of day (to minimize diurnal variation) at each specified time point: -D3, D0, D1, D3, D5, D7, D14, and D21.

**Immunocytochemistry staining**

Spinal cord samples were fixed in 4% PFA and then embedded in OCT compound for subsequent sectioning. Each tissue section was incubated with primary antibodies (GFAP, ABclonal, China, A19058; GAP-43, ABclonal, China, A16857) overnight at 4°C, followed by incubation with secondary antibodies (HRP Donkey Anti-Rabbit IgG (H+L), ABclonal, China, AS038; FITC Goat Anti-Rabbit IgG (H+L), ABclonal, China, AS011) away from light for 1 h at room temperature. Images were acquired via an LX7 fluorescence microscope (Olympus, Japan).

**Supplemental results**

**Table S1.** Correlation coefficients between various parameters and principal components (F1–F4)

|  | F1 | F2 | F3 | F4 |
| --- | --- | --- | --- | --- |
| Weight | 0.847 | 0.046 | 0.107 | 0.000 |
| BBB score | 0.980 | 0.004 | 0.004 | 0.012 |
| SLE  (hypointense) | 0.140 | 0.799 | 0.018 | 0.042 |
| SLE  (hyperintense) | 0.353 | 0.447 | 0.163 | 0.037 |
| Lesion size | 0.828 | 0.065 | 0.048 | 0.060 |
| Neuron density | 0.780 | 0.189 | 0.017 | 0.014 |
| TNF-α | 0.706 | 0.069 | 0.226 | 0.000 |
| IL-6 | 0.768 | 0.175 | 0.029 | 0.029 |
| IL-1β | 0.067 | 0.018 | 0.811 | 0.105 |
| IntDen/Area  (GFAP) | 0.967 | 0.031 | 0.001 | 0.001 |
| IntDen/Area  (GAP-43) | 0.381 | 0.340 | 0.197 | 0.082 |


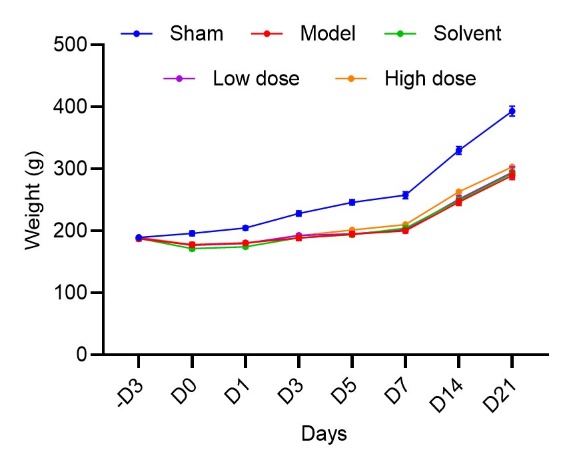


**Fig. S1** Changes in the body weights of the rats in the different groups over time. Body weights of rats in the Sham, Model, Solvent, Low-dose, and High-dose groups were recorded from –D3 to D21 at the indicated time points.

**
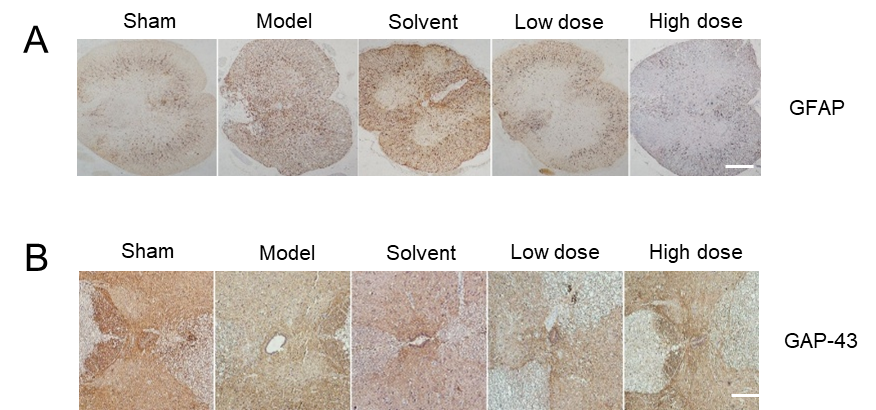
**

**Fig. S2** Immunohistochemical analysis of GFAP and GAP-43 expression in spinal cord tissues. (A) Immunohistochemical staining was performed to determine the expression levels of GFAP in spinal cord tissues from each group. (B) Immunohistochemical staining was performed to determine the expression levels of GAP-43 in spinal cord tissue sections from different treatment groups.


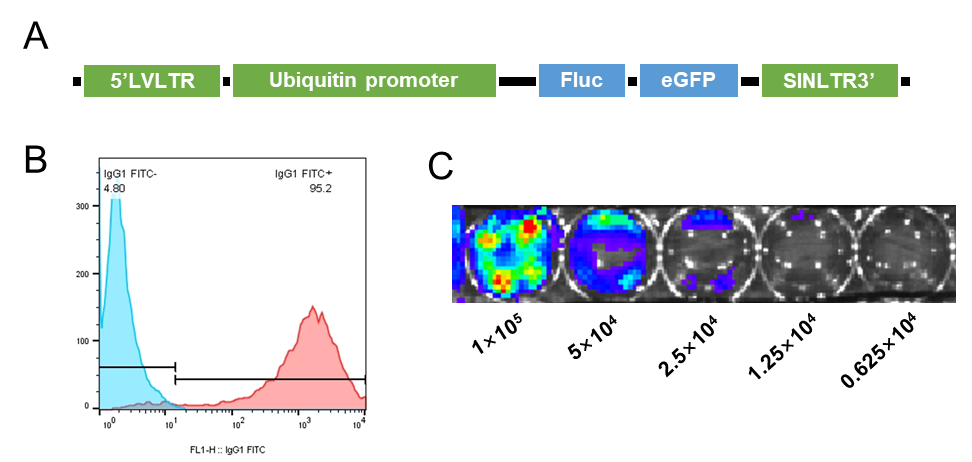


**Fig. S3** The dynamic metabolism of UC-MSCs *in vivo*. (**A**) Illustration of the lentivirus-mediated dual-fluorescence reporter construct (pLV-Fluc-eGFP). Fluc and GFP were used for BLI and green fluorescent protein imaging, respectively. (**B**) Flow cytometry analysis of UC-MSCs transduced with the lentivirus. The blue histogram represents the isotype control (IgG1 FITC-), and the red histogram shows the proportion of cells positive for the relevant marker (IgG1 FITC+), with 95.2% of the cells being positive, indicating efficient transduction. Live images of cultured UC-MSCs with luci (**C**) expression at a series of cell density gradients *in vitro*.


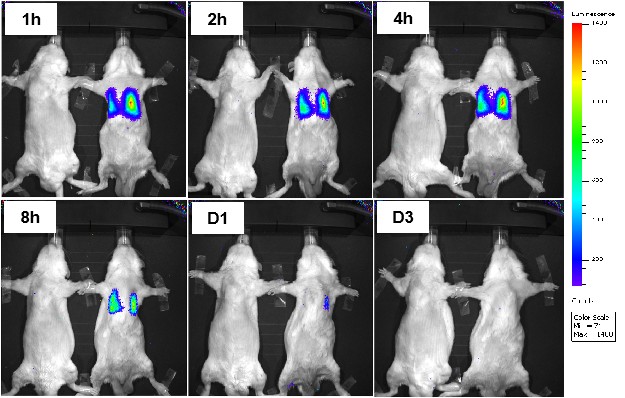


**Fig. S4** Determination of lung cell retention rate after intravenous injection of UC-MSCs. *In vivo* bioluminescence imaging of UC-MSCs in rats at 1 h, 2 h, 4 h, 8 h, D1, and D3 postadministration (left: negative control; right: UC-MSC-treated).
